# Cocaine Inhibits Dopamine D_2_ Receptor Signaling via Sigma-1-D_2_ Receptor Heteromers

**DOI:** 10.1371/journal.pone.0061245

**Published:** 2013-04-18

**Authors:** Gemma Navarro, Estefania Moreno, Jordi Bonaventura, Marc Brugarolas, Daniel Farré, David Aguinaga, Josefa Mallol, Antoni Cortés, Vicent Casadó, Carmen Lluís, Sergi Ferre, Rafael Franco, Enric Canela, Peter J. McCormick

**Affiliations:** 1 Centro de Investigación Biomédica en Red de Enfermedades Neurodegenerativas (CIBERNED) and Institute of Biomedicine of the University of Barcelona (IBUB) and Department of Biochemistry and Molecular Biology, Faculty of Biology, University of Barcelona, Barcelona, Spain; 2 National Institute on Drug Abuse, Intramural Research Program, National Institutes of Health, Department of Health and Human Services, Baltimore, Maryland, United States of America; 3 Centro de Investigación Médica Aplicada, Universidad de Navarra, Pamplona, Spain; University of Iowa, United States of America

## Abstract

Under normal conditions the brain maintains a delicate balance between inputs of reward seeking controlled by neurons containing the D_1_-like family of dopamine receptors and inputs of aversion coming from neurons containing the D_2_-like family of dopamine receptors. Cocaine is able to subvert these balanced inputs by altering the cell signaling of these two pathways such that D_1_ reward seeking pathway dominates. Here, we provide an explanation at the cellular and biochemical level how cocaine may achieve this. Exploring the effect of cocaine on dopamine D_2_ receptors function, we present evidence of σ_1_ receptor molecular and functional interaction with dopamine D_2_ receptors. Using biophysical, biochemical, and cell biology approaches, we discovered that D_2_ receptors (the long isoform of the D_2_ receptor) can complex with σ_1_ receptors, a result that is specific to D_2_ receptors, as D_3_ and D_4_ receptors did not form heteromers. We demonstrate that the σ_1_-D_2_ receptor heteromers consist of higher order oligomers, are found in mouse striatum and that cocaine, by binding to σ_1_ -D_2_ receptor heteromers, inhibits downstream signaling in both cultured cells and in mouse striatum. In contrast, in striatum from σ_1_ knockout animals these complexes are not found and this inhibition is not seen. Taken together, these data illuminate the mechanism by which the initial exposure to cocaine can inhibit signaling via D_2_ receptor containing neurons, destabilizing the delicate signaling balance influencing drug seeking that emanates from the D_1_ and D_2_ receptor containing neurons in the brain.

## Introduction

The striatum is the main input structure of the basal ganglia and consists of subcortical structures involved in the processing of information related with the performance and learning of complex motor acts and motivational processes and is altered in conditions such as Parkinson’s, Huntington’s and in drug addiction [1]. GABAergic striatal efferent neurons constitute more than 95% of the striatal neuronal population [2]. There are two major subtypes of GABAergic striatal efferent neurons: GABAergic dynorphinergic neurons, which express the peptide dynorphin and dopamine D_1_ receptors and GABAergic enkephalinergic neurons, which express the peptide enkephalin and dopamine D_2_ receptors [3]. In the case of drug addiction, and specifically cocaine, the dopaminergic pathway plays a critical role in the pathology [4], [5], specifically, the two populations of D_1_ and D_2_ containing neurons. These two pathways can control novelty seeking and reward-dependent learning as well as having opposite effects on motor activity [6]. Early studies performed in D_1_ receptor knockout mice showed the importance of dopamine D_1_ receptor in cocaine action as the activation of D_1_ receptors was an absolute requirement for the induction of the cellular and behavioral responses to cocaine [7]. In addition to opposing the locomotor effects of D_1_, D_2_ containing neurons also serve to oppose drug reinforcement [8]. In the context of cocaine it is known that the D_2_ is essential for cocaine’s effects [9] as D_2_ receptors are required to enhance the rewarding properties of cocaine [10]. In D_2_ −/− mutant animals the release of dopamine evoked by cocaine injection is dramatically higher compared to WT animals, and an intact D_2_-mediated signaling is required to elicit the rewarding and reinforcing effects of cocaine [11]. At the mechanistic level it was shown there is a switch from D_2_ to a D_1_ mediated increase on GABA_A_-IPSC in cocaine treated rats [12], and in models of long-term cocaine treatment it has been shown that D_1_ increases and D_2_ levels decrease [13]. Finally, it has been shown that the activation of postsynaptic D_2_ on striatopallidal neurons can facilitate drug reinforcement via inhibition of these neurons [8]. All of these studies point to a balance between D_1_ and D_2_ in controlling the motivational processes and reinforcement in drugs of abuse, and specifically cocaine.

The initial mechanistic steps of cocaine binding and its effects on these two striatal populations of neurons (D_1_ and D_2_ receptor containing neurons) are not well understood. What is known is cocaine is able to exert part of its behavioral and cellular effect by elevating dopamine levels in the striatum [14]. It achieves this by binding to and inhibiting the presynaptic dopamine transporter (DAT) [15]. Cocaine is a high-affinity inhibitor of DAT and upon binding to DAT cocaine causes a rapid increase in extracellular dopamine levels. Although DAT inhibition is required for cocaine’s effects, it is not the only required mechanism of action per the effects of D_1_ and D_2_ receptors discussed above. In fact, Cocaine is able to modulate dopamine signaling, via both the D_1_ and D_2_ family of dopamine receptors, which when activated can lead to stimulation or inhibition of signaling pathways. This provokes the question, how does cocaine seemingly influence two different receptor pathways? One potential answer lies in the fact that cocaine does not seem to bind the dopamine receptors directly but can bind to a receptor heteromer made up of the D_1_-like receptor family member, D_1_ and the σ_1_-receptor [16]. Through this latter interaction, cocaine can potentiate D_1_ receptor-mediated adenylyl cyclase activation, induce ERK1/2 phosphorylation and counteract the MAPK activation induced by D_1_ receptor stimulation [16]. However, as discussed above, D_2_ also plays a role in the early effects of cocaine. Here we explore the initial molecular events after cocaine exposure on the dopamine receptor D_2_ like family and test the hypothesis that σ_1_ receptor may provide the link between cocaine and the D_1_ and D_2_ receptor signaling balance.

## Materials and Methods

### Ethics Statement

The study received the approval of the Catalan Ethical Committee for Animal Use (CEAA/DMAH 4049 and 5664) and all procedures were performed to minimize animal suffering.

### Fusion Proteins and Expression Vectors

Sequences encoding amino acids residues 1–155 and 155–238 of YFP Venus protein, and amino acids residues 1–229 and 230–311 of RLuc8 protein were subcloned in pcDNA3.1 vector to obtain the YFP Venus (nVenus, cVenus) and RLuc8 (nRLuc8, cRLuc8) hemi-truncated proteins expressed in pcDNA3.1 vector. The human cDNA for the long isoform of dopamine D_2_ receptors (D_2_ receptors), adenosine A_2A_ or σ_1_ receptors cloned in pcDNA3.1 were amplified without their stop codons using sense and antisense primers harboring either unique *EcoRI* and *BamHI* sites (or *EcoRI* and *KpnI* sites for σ_1_ receptor). The fragments were then subcloned to be in-frame with Rluc, EYFP or GFP^2^ into the *EcoRI* and *BamHI* or *KpnI* restriction site of an Rluc-expressing vector (pRluc-N1, PerkinElmer, Wellesley, MA), an EYFP expressing vector (EYFP-N3; enhanced yellow variant of GFP; Clontech, Heidelberg, Germany) or an GFP^2^ expressing vector (GFP^2^-N2, Clontech) respectively, to give the plasmids that express receptors fused to either RLuc, YFP or GFP^2^ on the C-terminal end of the receptor (D_2_-RLuc, D_2_-YFP, D_2_-GFP^2^, σ_1_-Rluc, σ_1_-YFP, A_2A_-RLuc or A_2A_-YFP receptors respectively). The human cDNAs for D_2_ and σ_1_ receptors cloned in pcDNA3.1 were amplified without its stop codon using sense and antisense primers harboring unique *KpnI* and *EcoRI* sites to clone D_2_ and σ_1_ receptors in pcDNA3.1-cVenus, pcDNA3.1-nVenus, pcDNA3.1-cRLuc8 or pcDNA3.1-nRLuc8. The amplified fragments were subcloned to be in-frame with the multiple cloning sites of the vectors to give the plasmids that express D_2_ and σ_1_ receptors fused to either nVenus, cVenus, nRLuc8 or cRLuc8 on the C-terminal end of the receptor (D_2_-cVenus, D_2_-nVenus, D_2_-cRLuc8, D_2_-nRLuc8, σ_1_-nVenus, σ_1_-cVenus, σ_1_-nRluc8 or σ_1_-cRluc8, respectively). When analyzed by confocal microscopy, it was observed that all fusion proteins showed similar subcellular distribution than naïve receptors (see results and results not shown). Fusion of RLuc and YFP to D_2_ or A_2A_ receptors did not modify receptor function as previously determined by cAMP assays [17].

### Cell Culture and Chemical Reagents

HEK-293T cells were grown in Dulbecco’s modified Eagle’s medium (DMEM) supplemented with 2 mM L-glutamine, 100 U/ml penicillin/streptomycin, and 5% (v/v) heat inactivated Fetal Bovine Serum (FBS) (all supplements were from Invitrogen, Paisley, Scotland, UK). CHO cell lines were maintained in α-MEM medium without nucleosides, containing 10% fetal calf serum, 50 µg/mL penicillin, 50 µg/mL streptomycin, and 2 mM L-glutamine (300 µg/mL). Cells were maintained at 37°C in an atmosphere of 5% CO_2_, and were passaged when they were 80–90% confluent, i.e. approximately twice a week. HEK-293T or CHO cells were transiently transfected with the corresponding cDNAs by PEI (PolyEthylenImine, Sigma, St. Louis, MO, USA) method as previously described [18]or the corresponding siRNA by lipofectamine (Invitrogen™, Carlsbad, USA) method following the instructions of the supplier. siRNA that targets both human and rodent σ_1_ RNA and a scrambled control siRNA were purchased from Invitrogen (catalog HSS 145543). All ligands used are diagrammed in Figure S1. Cocaine-HCl was purchased from Spanish Agencia del Medicamento n°: 2003C00220. PD144418 and PRE were purchased from Tocris, Bristol, UK. Quinpirole and raclopride were purchased from Sigma, St. Louis, MO, USA.

### Immunocytochemistry

For immunocytochemistry, cells were fixed in 4% paraformaldehyde for 15 min and washed with PBS containing 20 mM glycine (buffer A) to quench the aldehyde groups. Then, after permeabilization with buffer A containing 0.2% Triton X-100 for 5 min, cells were treated with PBS containing 1% bovine serum albumin. After 1 h at room temperature, cells were labeled with the primary mouse monoclonal anti-Rluc receptor antibody (1/200, Millipore, CA, USA) or mouse monoclonal anti-σ_1_ receptor antibody (1/200; Chemicon) for 1 h, washed, and stained with the secondary Cy3 donkey anti-mouse antibody (1/200, Jackson Immunoresearch Laboratories, West Grove, PA, USA). D_2_ receptors fused to YFP protein were detected by their fluorescence properties. Samples were rinsed and observed in a Leica SP2 confocal microscope (Leica Microsystems, Mannheim, Germany).

### BRET and BRET with BiFC Assays

HEK-293T cells growing in six-well plates were transiently co-transfected with a constant amount of cDNA encoding for the receptor fused to RLuc or nRLuc8 and cRLuc8 proteins and with increasingly amounts of cDNA corresponding to the receptor fused to YFP or nVenus and cVenus proteins (see figure legends). To quantify receptor-YFP expression or receptor-reconstituted YFP Venus expression, cells (20 µg protein) were distributed in 96-well microplates (black plates with a transparent bottom) and fluorescence was read in a Fluoro Star Optima Fluorimeter (BMG Labtechnologies, Offenburg, Germany) equipped with a high-energy xenon flash lamp, using a 10 nm bandwidth excitation filter at 400 nm reading. Receptor-fluorescence expression was determined as fluorescence of the sample minus the fluorescence of cells expressing the BRET donor alone. For BRET or BRET with BiFC measurements, the equivalent of 20 µg of cell suspension were distributed in 96-well microplates (Corning 3600, white plates; Sigma) and 5 µM coelenterazine H (Molecular Probes, Eugene, OR) was added. After 1 minute for BRET or after 5 min for BRET with BiFC of adding coelenterazine H, the readings were collected using a Mithras LB 940 that allows the integration of the signals detected in the short-wavelength filter at 485 nm (440–500 nm) and the long-wavelength filter at 530 nm (510–590 nm). To quantify receptor-RLuc or receptor-reconstituted RLuc8 expression luminescence readings were also performed after 10 minutes of adding 5 µM coelenterazine H. Both fluorescence and luminescence of each sample were measured before every experiment to confirm similar donor expressions (about 150,000 luminescent units) while monitoring the increase acceptor expression (10,000–70,000 fluorescent units). The net BRET is defined as [(long-wavelength emission)/(short-wavelength emission)]-Cf where Cf corresponds to [(long-wavelength emission)/(short-wavelength emission)] for the donor construct expressed alone in the same experiment. BRET is expressed as mili BRET units, mBU (net BRET×1000).

### SRET Assays

HEK-293T cells growing in six-well plates were transiently co-transfected with constant amounts of cDNAs encoding for both receptor fused to RLuc and GFP^2^ proteins and with increasingly amounts of cDNA corresponding to the receptor fused to YFP protein and SRET was determined as previously described using a Mithras LB 40 [19].

### Striatal Slices Preparation

Brains from WT littermates and σ_1_ receptor KO CD1 albino Swiss male mice (8 weeks old, 25 g of weight) were generously provided by Laboratorios Esteve (Barcelona, Spain) [20]. Brains were rapidly removed from animals and striatal slices were obtained as previously indicated [16], [21].

### Coimmunoprecipitation

Striatal slices from WT littermates and σ_1_ receptor KO mice were treated with medium or with 150 µM cocaine for 30 min. The striatal tissue was disrupted with a Polytron homogenizer in 50 mM Tris-HCl buffer, pH 7.4, containing a protease inhibitor mixture (1/1000, Sigma). The cellular debris was removed by centrifugation at 13,000 g for 5 min at 4°C, and membranes were obtained by centrifugation at 105,000 g for 1 h at 4°C. Membranes were solubilized in ice-cold immunoprecipitation buffer (phosphate-buffered saline (PBS), pH 7.4, containing 1% (v/v) Nonidet P-40) and incubated for 30 min on ice before centrifugation at 105,000 g for 1 h at 4°C. The supernatant (1 mg/ml of protein) was processed for immunoprecipitation as described in the immunoprecipitation protocol using a Dynabeads® Protein G kit (Invitrogen) using goat anti-D_2_ receptor antibody (1:1000, Santa Cruz Biotechnology, Santa Cruz, CA). As negative control anti-FLAG antibody (1:1000, Sigma) was used. Protein was quantified by the bicinchoninic acid method (Pierce) using bovine serum albumin dilutions as standards. Immunoprecipitates were separated on a denaturing 10% SDS-polyacrylamide gel and transferred onto PVDF membranes. Membranes were blocked for 90 min in 5% Bovine (1% fat) dry milk and PBS-Tween 20 (0.05% V/V). The following primary antibodies were incubated overnight at 4°C in 5% milk and PBS-Tween 20 (0.05% V/V): mouse anti-D_2_ receptor antibody (1:1000, Santa Cruz Biotechnology, Santa Cruz, CA) or mouse anti-σ_1_ receptor antibody B-5 (sc-137075) (1:800, Santa Cruz Biotechnology, Santa Cruz, CA) and, after washing three times for 10 min in PBS Tween-20 (0.05% V/V), membranes were incubated with the secondary antibody rabbit anti-mouse-HRP (1:20,000, Dako, Glostrup, Denmark) for 1 h at room temperature in 5% milk and PBS-Tween 20 (0.05% V/V). After three washes with PBS Tween-20 (0.05% V/V) and a final wash with PBS, bands were detected with the addition of SuperSignal West Pico Chemiluminescent Substrate (Pierce) and visualized with a LAS-3000 (Fujifilm). Analysis of detected bands was performed by Image Gauge software (version 4.0) and Multi Gauge software (version 3.0).

### In Situ Proximity Ligation Assays (PLA)

Striatal slices from WT and σ_1_ receptor KO mice treated or not with 150 µM cocaine for 30 min, were mounted on slide glass and heteromers were detected using the Duolink II in situ PLA detection Kit (OLink; Bioscience, Uppsala, Sweden). Slices were thawed at 4°C, washed in 50 mM Tris-HCl, 0.9% NaCl pH 7.8 buffer (TBS), permeabilized with TBS containing 0.01% Triton X-100 for 10 min and successively washed with TBS. After 1 h incubation at 37°C with the blocking solution in a pre-heated humidity chamber, slices were incubated overnight in the antibody diluent medium with a mixture of equal amounts of the primary antibodies mouse anti-σ_1_ receptor antibody B-5 (sc-137075, 1:500, see above) and the guinea-pig anti-D_2_ receptor antibody (1:500 Sigma) which specificity for D_2_ receptors was previously demonstrated [21]. Slices were washed as indicated by the supplier and incubated for 2 h in a pre-heated humidity chamber at 37°C with PLA probes detecting mouse or guinea pig antibodies, Duolink II PLA probe anti-mouse plus and Duolink II PLA probe anti-guinea minus (prepared following the instructions of the supplier) diluted in the antibody diluent to a concentration of 1:5. After washing at room temperature, slices were incubated in a pre-heated humidity chamber for 30 min at 37°C, with the ligation solution (Duolink II Ligation stock 1:5 and Duolink II Ligase 1:40). Detection of the amplified probe was done with the Duolink II Detection Reagents Red Kit. After exhaustively washing at room temperature as indicated in the kit, slices were mounted using the mounting medium with DAPI. The samples were observed in a Leica SP2 confocal microscope (Leica Microsystems, Mannheim, Germany). Images were opened and processed with Image J confocal.

### Immunohistochemistry

Striatal slices from WT and σ_1_ receptor KO mice were thawed at 4°C, washed in TBS, permeabilized with TBS containing 0.1% Triton X-100 for 10 min and successively washed with TBS. Slices were rocked in Blocking reagent 1% (Roche, Sant Cugat del Vallés, Spain) for 1 h at 37°C in a humidified atmosphere and incubated overnight at 4°C in a humidified atmosphere with the primary antibodies: mouse anti-σ_1_ receptor antibody B-5 (sc-137075, 1:100, see above) or the guinea-pig anti-D_2_ receptor antibody (1:100 Frontier Institute, Ishikari, Hokkaido, Japan), in 0.1% TBS-Tween, 0.1% BSA-Acetylated (Aurion, Wageningen, The Netherlands), 7% SND. Slices were washed in TBS-Tween 0.05% and left for 2 h at room temperature in a humidified atmosphere with the corresponding secondary antibodies: goat anti-mouse (1:200, Alexa Fluor 488, Invitrogen) and goat anti-guinea pig (1:200, Alexa Fluor 488, Invitrogen) in the same medium. Then, the slices were washed in TBS-Tween 0.05%, followed by a single wash in TBS before mounting in Mowiol medium (Calbiochem, Merck, Darmstadt, Germany), covered with a glass and left to dry at 4°C for 24 h. The sections were observed and imaged in a Leica SP2 confocal microscope.

### cAMP Determination

Non transfected or transiently transfected CHO cells (see figure legends) were treated for 10 min with the indicated concentrations of D_2_ receptor agonist quinpirole, 30 µM cocaine or 100 nM of the σ_1_ receptor agonist PRE-084 alone or in combination. cAMP production was determined using [^3^H]cAMP kit (Amersham Biosciences, Uppsala, Sweden) following the instructions from the manufacturer.

### ERK 1/2 Phosphorylation Assays

WT and KO ice striatal slices were treated for the indicated time with the indicated concentrations of cocaine and/or D_2_ receptor ligands, frozen on dry ice and stored at −80°C. When ERK1/2 phosphorylation assays were performed in cell cultures, CHO cells (48 h after transfection) were cultured in serum-free medium for 16 h before the addition of the indicated concentration of cocaine or/and D_2_ receptor ligands for the indicated time. Both, cells and slices were lysed in ice-cold lysis buffer (50 mM Tris-HCl pH 7.4, 50 mM NaF, 150 mM NaCl, 45 mM β-glycerophosphate, 1% Triton X-100, 20 µM phenyl-arsine oxide, 0.4 mM NaVO_4_ and protease inhibitor cocktail) and ERK 1/2 phosphorylation was determined as indicated elsewhere [16], [22].

### CellKey Label-free Assays

The CellKey system provides a universal, label-free, cell-based assay platform that uses cellular dielectric spectroscopy (CDS) to measure endogenous and transfected receptor activation in real time in live cells [23]. Changes in the complex impedance (DZ or dZ) of a cell monolayer in response to receptor stimulation were measured. Impedance (Z) is defined by the ratio of voltage to current as described by Ohm’s law (Z = V/I). CHO cell clones stably expressing D_2_ receptors were grown to confluence in a CellKey Standard 96 well microplate that contains electrodes at the bottom of each well. For untreated cells or for cells preincubated (overnight at 37°C) with PTx (10 ng/ml), medium was replaced by HBSS buffer (Gibco) supplemented with 20 mM HEPES 30 minutes prior to running the cell equilibration protocol. A baseline was recorded for 5 minutes and then cells were treated with increasing concentrations of the D_2_ receptor agonist quinpirole or cocaine alone or in combination and data was acquired for the following 10 minutes. To calculate the impedance, small voltages at 24 different measurement frequencies were applied to treated or non-treated cells. At low frequencies, extracellular currents (iec) that pass around individual cells in the layer were induced. At high frequencies, transcellular currents (itc) that penetrate the cellular membrane were induced and the ratio of the applied voltage to the measured current for each well is the impedance. The data shown refer to the maximum complex impedance induced extracellular currents (Ziec) response to the ligand addition.

## Results

### σ_1_ Receptors form Heteromers with Dopamine D_2_ Receptors but not with the Other D_2_-like Receptor Family Members

**Figure 1:**
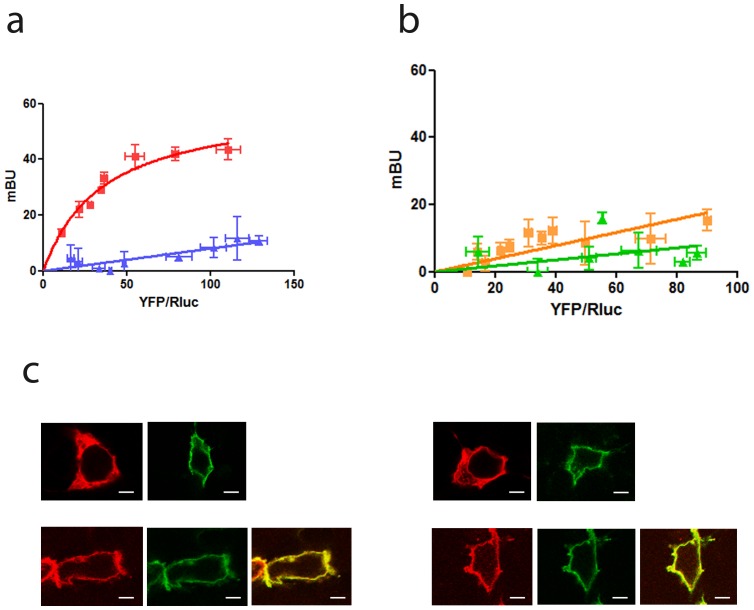
Molecular interaction between σ_1_ receptors and D_2_ receptors in living cells. BRET saturation experiments were performed with HEK-293T cells co-transfected with: (**a**) D_2_-RLuc cDNA (0.4 µg, squares) or adenosine A_2A_-RLuc cDNA as negative control (0.2 µg, triangles) and increasing amounts of σ_1_-YFP cDNA (0.1 to 1 µg cDNA), (**b**) D_3_-RLuc cDNA (0.5 µg, squares) or D_4_-RLuc cDNA (0.5 µg, triangles) and increasing amounts of σ_1_-YFP cDNA (0.1 to 1 µg cDNA). The relative amount of BRET acceptor is given as the ratio between the fluorescence of the acceptor minus the fluorescence detected in cells only expressing the donor, and the luciferase activity of the donor (YFP/Rluc). BRET data are expressed as means ± S.D. of five to six different experiments grouped as a function of the amount of BRET acceptor. In (**c**) confocal microscopy images of HEK-293T cells transfected with D_2_-YFP or σ_1_-RLuc (top panels) or co-transfected with D_2_-YFP and σ_1_-RLuc (bottom panels), treated (right images) or not (left images) with 30 µM cocaine for 30 min. σ_1_ receptors (red) were identified by immunocytochemistry and D_2_ receptors (green) were identified by its own fluorescence. Co-localization is shown in yellow. Scale bar:10 µm.

We first examined whether the receptors of the D_2_-like family could directly interact with σ_1_ receptors and thus be a target for cocaine binding. To do this we used the Bioluminescence Resonance Energy Transfer (BRET) technology in HEK-293T cells expressing a constant amount of D_2_ (long isoform), D_3_ or D_4_ dopamine receptors fused to *Renilla Luciferase* (RLuc) and increasing amounts of σ_1_ receptors fused to Yellow Fluorescence Protein (YFP). Clear BRET saturation curves were obtained in cells expressing D_2_-RLuc receptors and increasing amounts of σ_1_-YFP receptors with a BRET_max_ of 55±7 mBU and a BRET_50_ of 28±6 (Fig. 1a). In contrast, in cells expressing D_3_-RLuc or D_4_-RLuc and σ_1_-YFP receptors a low and linear non-specific BRET signal was obtained thus confirming the specificity of the interaction between D_2_-RLuc and σ_1_-YFP receptors (Fig. 1b). As a further control, cells were cotransfected with σ_1_-YFP receptors and adenosine A_2A_-Rluc receptors and no specific BRET signal was obtained (Fig. 1a). These results indicate that σ_1_ receptors selectively interact with dopamine D_2_ receptors and not with the other members of the D_2_-like receptor family.

The σ_1_ receptors are predominantly found in the endoplasmic reticulum membrane and the plasma membrane [24] with one hypothesis that it may be acting as a chaperone protein [25]. The expression of σ_1_ and D_2_ receptors at the plasma membrane level was explored by analyzing the co-localization of both receptors by confocal microscopy. HEK-293T cells were used in the assays since they constitutively express σ_1_ receptors, but not DAT [16]. As expected, a punctate σ_1_ receptor staining in naïve (Fig. 1c left panels, top images) or cocaine-treated (Fig. 1c right panels, top images) HEK-293T cells was detected. After transfection of the cDNA corresponding to D_2_ receptors, a co-localization of σ_1_ receptor and D_2_ receptors was detected at the plasma membrane level in cells not treated with cocaine (Fig. 1c left panels, bottom images) or in cells treated with 30 µM cocaine for 30 min (Fig. 1c right panels, bottom images).

### Higher Order Complex Formation between σ_1_ Receptors and Dopamine D_2_ Receptors

**Figure 2:**
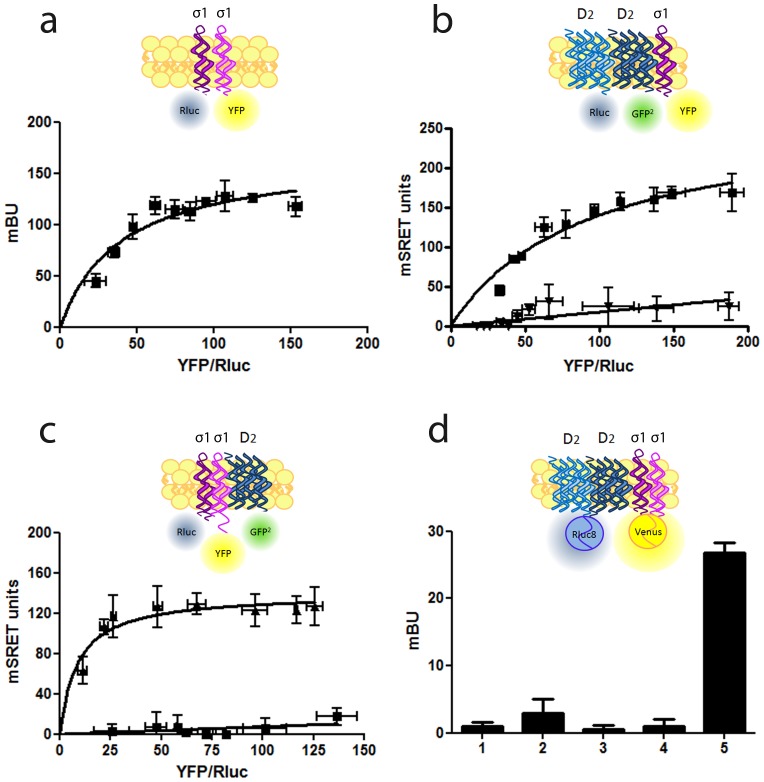
Higher order complex formation between σ_1_ receptors and dopamine D_2_ receptors in living cells. In (**a**) BRET saturation experiments were performed with HEK-293T cells co-transfected with σ_1_-RLuc cDNA (0.2 µg) and increasing amounts of σ_1_-YFP cDNA (0.1 to 0.6 µg cDNA). A schematic representation of a BRET process is shown at top in which the receptor fused to RLuc acts as donor and the receptor fused to YFP acts as acceptor. In (**b**) and (**c**) SRET saturation experiments were performed with HEK-293T cells co-transfected with: (b) a constant amount of D_2_-RLuc (0.6 µg) and D_2_-GFP^2^ (1 µg) receptor cDNA (squares) or A_2A_-RLuc (0.3 µg) and A_2A_-GFP^2^ (0.5 µg) receptor cDNA, as negative control (triangles), and increasing amounts of σ_1_-YFP receptor (0.2 to 1.5 µg cDNA), (c) a constant amount of σ_1_-Rluc (0.3 µg) and D_2_-GFP^2^ (1 µg) (triangles) or A_2_-GFP^2^ (0.5 µM) as negative control (squares) receptor cDNA and increasing amounts of σ_1_-YFP receptor cDNA (0.2 to 1.5 µg). The relative amount of acceptor is given as the ratio between the fluorescence of the acceptor minus the fluorescence detected in cells only expressing the donor, and the luciferase activity of the donor (YFP/Rluc). A schematic representation of a SRET process is shown at top images in which two sequential energy transfer events between Rluc and GFP^2^ (BRET process) and between GFP^2^ and YFP (FRET process) occurs. In (**d**) BRET with luminescence/fluorescence complementation approach was performed measuring BRET in cells co-transfected with 1 µg of the two cDNAs corresponding to D_2_-nRLuc8 and D_2_-cRLuc8 and with 1.5 µg of the two cDNAs corresponding to σ_1_-nVenus and σ_1_-cVenus (5). As negative controls, cells transfected with the same amount of cDNA corresponding to D_2_-nRLuc8, D_2_-cRLuc8, σ_1_-nVenus and cVenus (1), D_2_-nRLuc8, D_2_-cRLuc8, σ_1_-cVenus and nVenus (2), D_2_-nRLuc8, σ_1_-nVenus, σ_1_-cVenus and cRLuc8 (3), or D_2_-cRLuc8, σ_1_-nVenus, σ_1_-cVenus and nRLuc8 (4) did not display any significant luminescence or positive BRET. A schematic representation of a BRET with luminescence/fluorescence complementation approach is given at the top image in which one receptor fused to the N-terminal fragment (nRluc8) and another receptor fused to the C-terminal fragment (cRluc8) of the Rluc8 act as BRET donor after Rluc8 reconstitution by a close receptor-receptor interaction and one receptor fused to an YFP Venus N-terminal fragment (nVenus) and another receptor fused to the YFP Venus C-terminal fragment (cVenus), act as BRET acceptor after YFP Venus reconstitution by a close receptor-receptor interaction. BRET or SRET data are expressed as means ± S.D. of five to six different experiments grouped as a function of the amount of BRET or SRET acceptor.

Recent crystal structures have demonstrated that homodimers of GPCRs are possible, a fact that has been confirmed for dopamine D_2_ receptors [26]–[30]. Considering that σ_1_ may act as a chaperone like molecule we investigated the possible formation of higher order receptor complexes between σ_1_ and D_2_ receptor homomers. To test this we first needed to know whether σ_1_-receptors could form dimers, something that had not been reported. First, we tested if σ_1_ receptors can form dimers by BRET experiments in HEK-293T cells expressing a constant amount of σ_1_-RLuc receptors and increasing amounts of σ_1_-YFP receptors. A positive and saturable BRET signal was obtained with a BRET_max_ of 165±35 mBU and a BRET_50_ of 22±12 (Fig. 2a) indicating that σ_1_-σ_1_ homodimers can exist and demonstrating, for the first time, the oligomerization of σ_1_ receptors. Next, we tested whether D_2_ receptor homomers could interact with σ_1_-receptors by a combined BRET and FRET assay termed Sequential Resonance Energy Transfer (SRET) [19]. This assay involves two sequential energy transfer events, one bioluminescent energy transfer between Rluc and a blue shifted GFP^2^ and a second fluorescent energy transfer event between excited GFP^2^ and YFP (see Fig. 2b top scheme). In HEK-293T cells expressing a constant amount of D_2_-RLuc and D_2_-GFP^2^ receptors and increasing amounts of σ_1_-YFP receptors, a net SRET saturation curve was obtained with a SRET_max_ of 269±33 SU and a SRET_50_ of 92±24 (Fig. 2b). Cells expressing constant amounts of adenosine A_2A_-RLuc and A_2A_-GFP^2^ receptors and increasing amounts of σ_1_-YFP receptors provided very low and linear SRET, according to the lack of interaction between A_2A_ receptors and σ_1_ receptors. These results demonstrate that σ_1_ receptors are able to form heteromers with D_2_-D_2_ receptor homomers. A net SRET saturation curve was also obtained using HEK-293T cells expressing constant amounts of σ_1_-Rluc and D_2_-GFP^2^ and increasing amounts of σ_1_-YFP (SRET_max_: 140±8 SU; SRET_50_: 9±3) but not when D_2_-RLuc and D_2_-GFP^2^ receptors were replaced by A_2A-_RLuc and A_2A_-GFP^2^ receptors (Fig. 2c). These results demonstrate that D_2_ receptors are able to form heteromers with σ_1_-σ_1_ receptor homomers. Finally, we tested for a higher order interaction of receptor heteromers constituted by σ_1_ and D_2_ receptor homomers (σ_1_-σ_1_-D_2_-D_2_). This was done using a modified BRET assay that involves a double complementation assay [30]. A diagram showing the BRET with luminescence/fluorescence complementation approach (BRET with BiFC assay; see Methods) is shown in Figure 2d (top panel). Briefly, one receptor fused to the N-terminal fragment (nRluc8) and another receptor fused to the C-terminal fragment (cRluc8) of the Rluc8 act as BRET donor after Rluc8 reconstitution by a close receptor-receptor interaction and one receptor fused to an YFP Venus N-terminal fragment (nVenus) and another receptor fused to the YFP Venus C-terminal fragment (cVenus), act as BRET acceptor after YFP Venus reconstitution by a close receptor-receptor interaction. Accordingly, cells were co-transfected with a constant amount of the two cDNAs corresponding to D_2_-nRLuc8 and D_2_-cRLuc8 (equal amounts of the two cDNAs) and with a constant amount of the two cDNAs corresponding to σ_1_-nVenus and σ_1_-cVenus (equal amounts of the two cDNAs). Specific BRET would only be possible if RLuc reconstituted by D_2_-nRLuc8-D_2_-cRLuc8 dimerization is close enough to YFP Venus reconstituted by σ_1_-nVenus-σ_1_-cVenus dimerization. Higher order heterotetramers were in fact observed as evidenced by a positive BRET signal (Fig. 2d). As negative controls, cells expressing only three fusion proteins and the fourth receptor not fused provided neither a significant fluorescent signal nor a positive BRET (Figure 2d). Collectively these results indicate that σ_1_-D_2_ receptor heteromers seem to be constituted by the interaction of receptor homomers and the minimal structural unit is the σ_1_-σ_1_-D_2_-D_2_ receptor heterotetramer.

### The Effect of σ_1_ Receptor Ligands on σ_1_-D_2_ Receptor Heterotetramer

**Figure 3:**
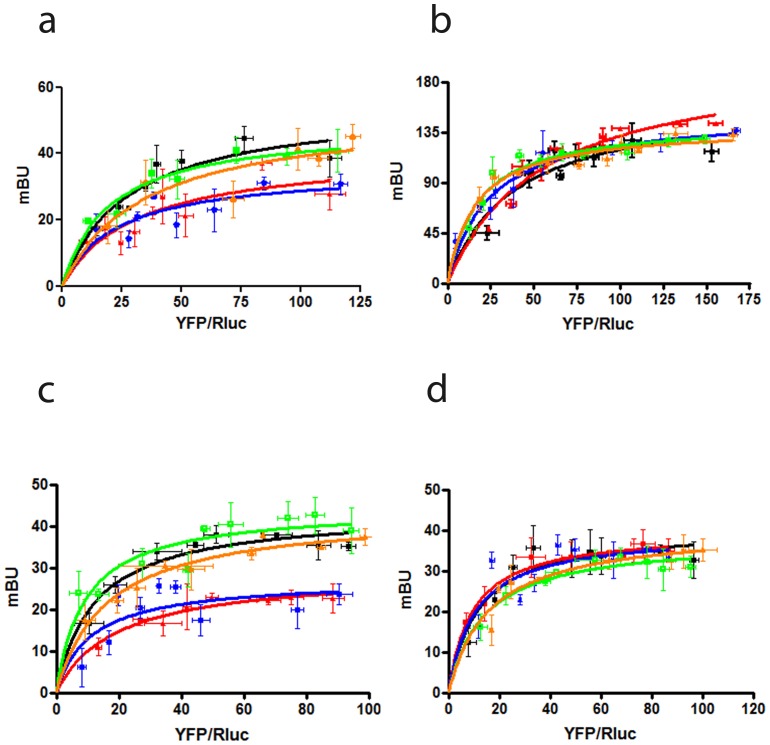
Effect of σ_1_ receptor ligands on σ_1_-D_2_ receptor heteromer. BRET was measured in HEK-293T cells cotransfected with: (**a**) D_2_–Rluc cDNA (0.4 µg) and increasing amounts of σ_1_-YFP receptor cDNA (0.1 to 1 µg), (**b**) σ_1_–Rluc cDNA (0.2 µg) and increasing amounts of σ_1_-YFP receptor cDNA (0.1 to 1 µg), (**c**) D_2_–Rluc cDNA (0.4 µg) and increasing amounts of D_2_-YFP receptor cDNA (0.2 to 2 µg) or (**d**) siRNA corresponding to σ_1_ receptor (see Methods), D_2_–Rluc cDNA (0.4 µg) and increasing amounts of D_2_-YFP receptor cDNA (0.2 to 2 µg)**,** not treated (black), treated for 30 min with 30 µM cocaine (red), treated for 10 min with 100 nM PRE084 (blue) or 1 µM PD144418 (green) or treated for 30 min with 30 µM cocaine and 1 µM PD144418 (orange)**.** The relative amount of BRET acceptor is given as the ratio between the fluorescence of the acceptor minus the fluorescence detected in cells only expressing the donor, and the luciferase activity of the donor (YFP/Rluc). BRET data are expressed as means ± SD of four to six different experiments grouped as a function of the amount of BRET acceptor.

It is known that cocaine can bind to σ_1_ [25], [31], [32]. We sought to measure the effect of cocaine binding to σ_1_ receptors on σ_1_-D_2_ receptor heteromers using BRET. We performed BRET experiments in HEK-293T cells expressing a constant amount of D_2_-RLuc receptors and increasing amounts of σ_1_-YFP receptors in the presence or in the absence of cocaine. The BRET saturation curve was reduced when cells were treated for 30 min with 30 µM of cocaine (BRET_max_: 35±6 mBU; BRET_50_: 26±8) indicating that cocaine binding to σ_1_ receptors induces structural changes in the σ_1_-D_2_ receptor heteromer. The cells treated (10 min) with the σ_1_ agonist PRE084 (100 nM; BRET_max_: 40±8 mBU; BRET_50_: 31±6) but not with the antagonist PD144418 (1 µM; BRET_max_: 48±3 mBU; BRET_50_: 20±5) also showed a decrease in the BRET saturation curves. Interestingly, the σ_1_ antagonist PD144418 is able to revert the effect induced by cocaine (BRET_max_: 52±9 mBU; BRET_50_: 31±7 in the presence of cocaine and PD144418) (Fig. 3a). To know if structural changes in σ_1_-σ_1_ receptor homomers or in D_2_-D_2_ receptor homomers can account for the ligand-induced effect on σ_1_-D_2_ receptor heteromers, we performed BRET experiments first in cells expressing σ_1_-RLuc and σ_1_–YFP receptors as indicated in Fig. 2a. Cells were treated for 10 min with 100 nM of the agonist PRE084 or 1 µM of the antagonist PD144418 or for 30 min with 30 µM of cocaine alone or with 1 µM PD144418. As shown in Fig. 3b, no significant changes in BRET_max_ or BRET_50_ were observed. Then, changes in the BRET saturation curve obtained in cells expressing a constant amount of D_2_-RLuc receptors and increasing amounts of D_2_-YFP receptors (BRET_max_: 44±3 mBU; BRET_50_: 12±4) were analyzed. The BRET saturation curve changed in cells treated for 10 min with 100 nM of PRE084 (BRET_max_: 27±5 mBU; BRET_50_: 11±4) or 30 min with 30 µM of cocaine (BRET_max_: 29±2 mBU; BRET_50_: 19±5) but not in cells treated for 10 min with 1 µM of PD144418 (BRET_max_: 44±3 mBU; BRET_50_: 9±3). Again the antagonist, PD144418, was able to revert the effect induced by cocaine (BRET_max_: 43±2 mBU; BRET_50_: 16±3 in cells pretreated with PD144418 and cocaine) (Fig. 3c). These data suggest structural changes in the complex brought about by binding of either the σ_1_ agonist PRE084 or cocaine. To test whether the effect of PRE084 or cocaine on D_2_-D_2_ heteromers are due to the presence of σ_1_ receptors, assays were performed in cells whose σ_1_ receptor expression was knocked-down using an RNAi approach (Fig. 3d). When we transfected a specific small interfering RNA (siRNA), a robust silencing of σ_1_ receptor expression was obtained (Fig. S2). The treatment with the specific siRNA completely abolished the effect of cocaine or PRE084 on the BRET saturation curve. The treatment with PD144418 or PD144418 and cocaine had no effect on these knocked-down cells (Fig. 3d). These results suggest that ligand binding to σ_1_ receptors induces strong changes in the structure of the D_2_-D_2_ receptor homomers in the σ_1_-D_2_ receptor heteromers.

### Cocaine Binding to σ_1_ Receptors Modulates the D_2_ Receptor Signaling in Transfected Cells

**Figure 4:**
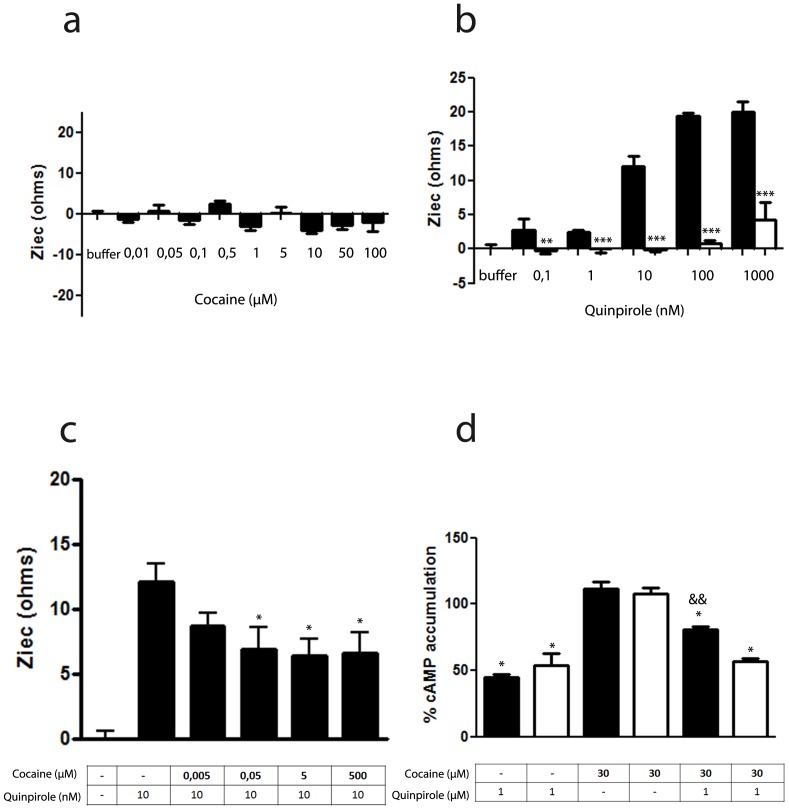
Cocaine binding to σ_1_ receptor modulates the G_i_-dependent D_2_ receptor signaling in transfected cells. In (**a** to **c**) CellKey label-free assays were performed in CHO cells stable expressing D_2_ receptors. In (a) cells were stimulated with buffer (B) or with increasing concentrations of cocaine. In (b) cells were preincubated (black columns) or not (white columns) with PTx (10 ng/ml) overnight and stimulated with buffer (B) or increasing concentrations of quinpirole. In (c) cells were stimulated with increasing concentrations of cocaine in the presence of 10 nM of quinpirole. In (d) cAMP production was determined in CHO cells stable expressing D_2_ receptors not transfected (black columns) or transfected (white columns) with siRNA corresponding to σ_1_ receptor (6.25 µg of oligonucleotides) and stimulated with 5 µM forskolin in absence (100%) or presence of 1 µM quinpirole, 30 µM cocaine alone or in combination. Percent of cAMP produced respect to 5 µM forskolin treatment was represented. Results are as mean ± S.E.M from 4–8 independent experiments. Statistical significance was calculated by one way ANOVA followed by Bonferroni multiple comparison test; in b **p<0.01 and ***p<0.005 compared with cells not transfected with siRNA, in c *p<0.05 compared with cells only treated with quinpirole, in d ^&&^p<0.01 compared to the corresponding quinpirole-treated cells and *p<0.05 and ***p<0.005 compared with forskolin-treated cells (100%).

The cocaine-induced modifications of the quaternary structure of D_2_ receptor homodimers in the σ_1_-D_2_ receptor heteromer described above suggest that cocaine can modulate the functionality of D_2_ receptors. To study how cocaine affects D_2_ receptor-mediated signaling, Chinese hamster ovary (CHO) cells were used as they provided a lower baseline of signaling for which to detect downstream changes and have been shown to constitutively express σ_1_ receptors but not DAT [16]. The effect of cocaine on D_2_ receptor agonist-induced, G protein-mediated signaling was measured using a label free assay that measures changes in cell impedance in response to stimulation. In CHO cells stably expressing D_2_ receptors, increasing cocaine concentrations (10 nM to 100 µM) did not give any G protein-mediated signaling, neither G_i/0_, G_S_ or G_q_ (Fig. 4a) as compared to known control receptors (Fig. S3). The signaling obtained upon D_2_ receptor activation with the agonist quinpirole (0.1 nM to 1 µM) showed a G_i_ profile (increases in impedance) that was completely blocked when cells were treated with the G_i_ specific pertussis toxin (PTx) (Fig. 4b). We observed a small but significant decrease in the G_i_ activation induced by quinpirole when cells where pre-treated for 1 h with 30 µM cocaine (Fig. 4c). These results indicate that cocaine by itself is not able to induce a G protein-mediated signaling but can partially inhibit the ability of D_2_ receptors to signal through G_i_. A downstream consequence of G_i_ mediated signaling is the ability to decrease cAMP signaling. In addition to the label free experiments above we determined the levels of cAMP in CHO cells stably expressing D_2_ receptors using forskolin and then measured whether cocaine was able to decrease the forskolin-induced cAMP formation. We found cocaine alone could not decrease the levels of cAMP after treatment with forskolin compared to the D_2_ agonist quinpirole (Fig. 4d). However, cocaine significantly dampened the quinpirole-induced decreases of forskolin-mediated increases in cAMP levels (Fig. 4d). This effect was blocked when cells were transfected with siRNA against the σ_1_ receptor (Fig. 4d), demonstrating that cocaine’s ability to counteract the action of quinpirole was mediated by σ_1_ receptors. Similar results were obtained when instead of cocaine the σ_1_ receptor agonist PRE084 was used (Fig. S4) reinforcing the concept that σ_1_ receptor ligands induce a significant decrease in the ability of D_2_ receptors to signal through G_i_.

Apart from G protein-mediated signaling, many GPCRs are able to signal in a G protein-independent way [33]–[37]. ERK 1/2 phosphorylation is one of the MAPK pathways that has been described to be activated in a G protein-independent and arrestin-dependent mechanism [36]. Several reports have highlighted the importance of ERK 1/2 activation in D_2_ receptors containing neurons for the effects of cocaine [38]–[41]. We sought to understand how cocaine might influence σ_1_-D_2_ receptor heteromer-mediated ERK 1/2 signaling. Varying concentrations of cocaine and varying the time of treatment did not lead to any significant change in ERK 1/2 phosphorylation in response to cocaine in cells not expressing D_2_ receptors (Fig. S5). Importantly, cocaine *per se* dose-dependently (Fig. S6a) and time-dependently (Fig. S6b) activated ERK 1/2 phosphorylation in cells expressing D_2_ receptors. This effect was mediated by σ_1_ receptors since it was strongly diminished in cells transfected with the σ_1_ receptors siRNA (Figs. S6a and S6b). The D_2_ receptor agonist quinpirole was also dose-dependently (Fig. S6c) and time-dependently (Fig. S6d) able to activate ERK 1/2 phosphorylation but, as expected, this effect was not mediated by σ_1_ receptors since it was not diminished in cells transfected with the σ_1_ receptors siRNA (Figs. S6c and S6d). These results point out that σ_1_ or D_2_ receptor activation in the σ_1_-D_2_ receptor heteromer induces ERK 1/2 phosphorylation. Thus, cocaine, like quinpirole, can act as an agonist at the MAPK activation level for the heteromer.

**Figure 5:**
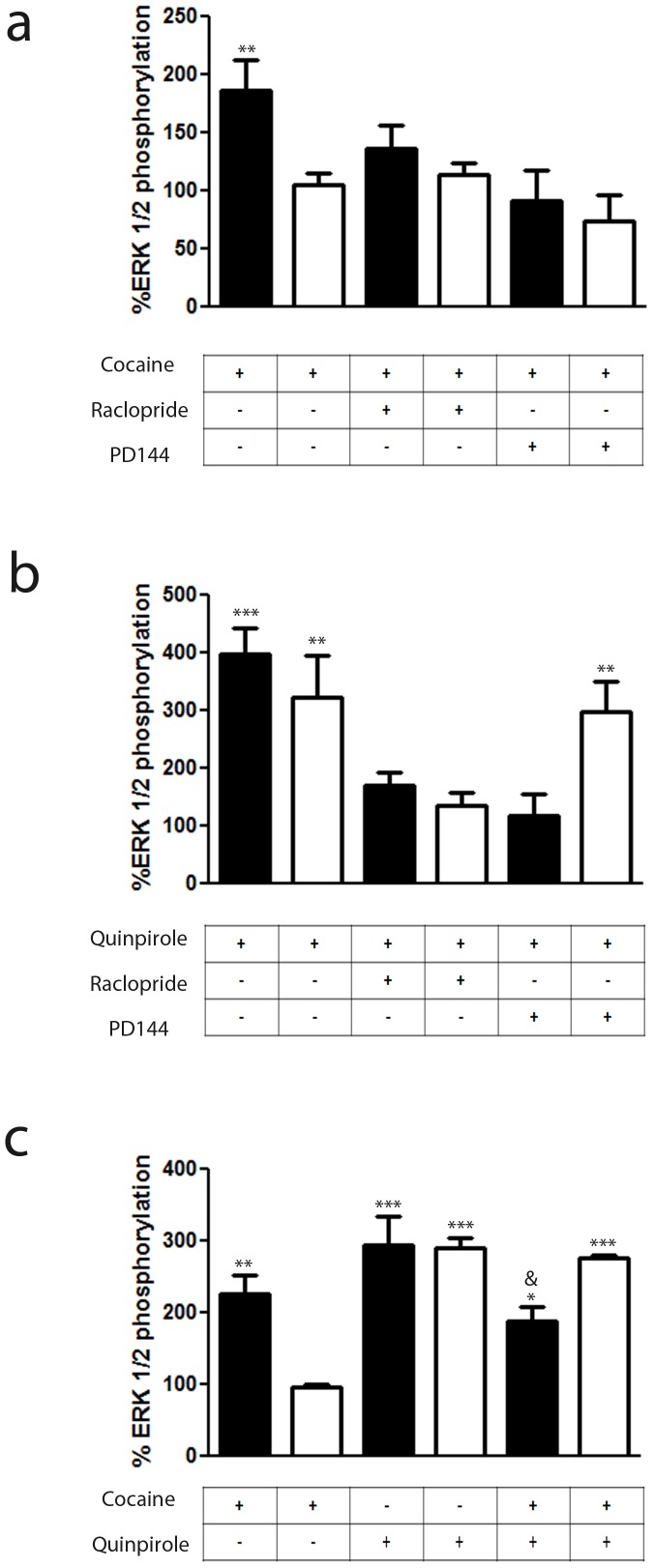
Cocaine binding to σ_1_ receptor modulates the ERK 1/2 signaling in transfected cells. CHO cells were transfected with D_2_ receptor cDNA (1 µg, black bars) or cotransfected (white bars) with D_2_ receptor cDNA and σ_1_ receptor siRNA (6.25 µg of oligonucleotides). Cells were incubated for 30 min (a) or 10 min (b) with medium (basal) or with 30 µM cocaine (a) or 1 µM quinpirole (b) in the absence or in the presence of 10 µM raclopride or 100 nM PD144418. In (**c**) cells were treated with medium (basal), 30 µM cocaine for 30 min, 1 µM quinpirole for 10 min or 30 µM cocaine for 30 min and, during the last 10 min, with 1 µM quinpirole. In all cases, ERK 1/2 phosphorylation is represented as percentage over basal levels (100%). Results are mean ± SEM of six to eight independent experiments performed in duplicate. Bifactorial ANOVA showed a significant (**p<0.01 and ***P<0.005) effect over basal.

A property of some receptor heteromers is the ability of the antagonist of one receptor to block the function of the agonist of the partner receptor, a property defined as cross-antagonism [22], [42]. In cells expressing D_2_ receptors we looked for cross-antagonism among σ_1_-D_2_ receptor heteromers. Indeed we found the cocaine-induced ERK 1/2 phosphorylation was counteracted not only by the σ_1_ receptor antagonist PD144418 (1 µM) but also by the D_2_ receptor antagonist raclopride (10 µM) (Fig. 5a). Analogously, the D_2_ receptor agonist quinpirole-induced ERK 1/2 phosphorylation was blocked by raclopride but also by PD144418 (Fig. 5b). These data suggest that antagonist binding leads to structural changes within the receptor heteromer that block signaling through the partner receptor. By definition an antagonist cannot signal on its own, therefore this cross-antagonism can only derive from the direct protein-protein interactions established between the receptors in the σ_1_-D_2_ receptor heteromer. This hypothesis is further supported by the fact that silencing cells of the σ_1_ receptor led to a complete loss in this cross-antagonism. That is, the effect of PD144418 on quinpirole-induced ERK1/2 phosphorylation was not observed when cells were transfected with the siRNA for σ_1_ receptors (Fig. 5b).

As mentioned above cocaine can inhibit DAT and increase the dopamine concentration in the striatum; so, in the presence of cocaine both receptors in the σ_1_-D_2_ receptor heteromer could be activated. Therefore we asked, what happens to ERK 1/2 phosphorylation after co-activation of both receptors? Surprisingly, a negative cross-talk was detected. When cells expressing D_2_ receptors were treated with both 1 µM quinpirole and 30 µM cocaine there was a decrease in ERK 1/2 phosphorylation compared to quinpirole alone (Fig. 5c). This difference was not seen if the cells were depleted of σ_1_ receptors via siRNA (Fig. 5c).

### σ_1_-D_2_ Receptor Heteromers are Found in the Brain Striatum

**Figure 6:**
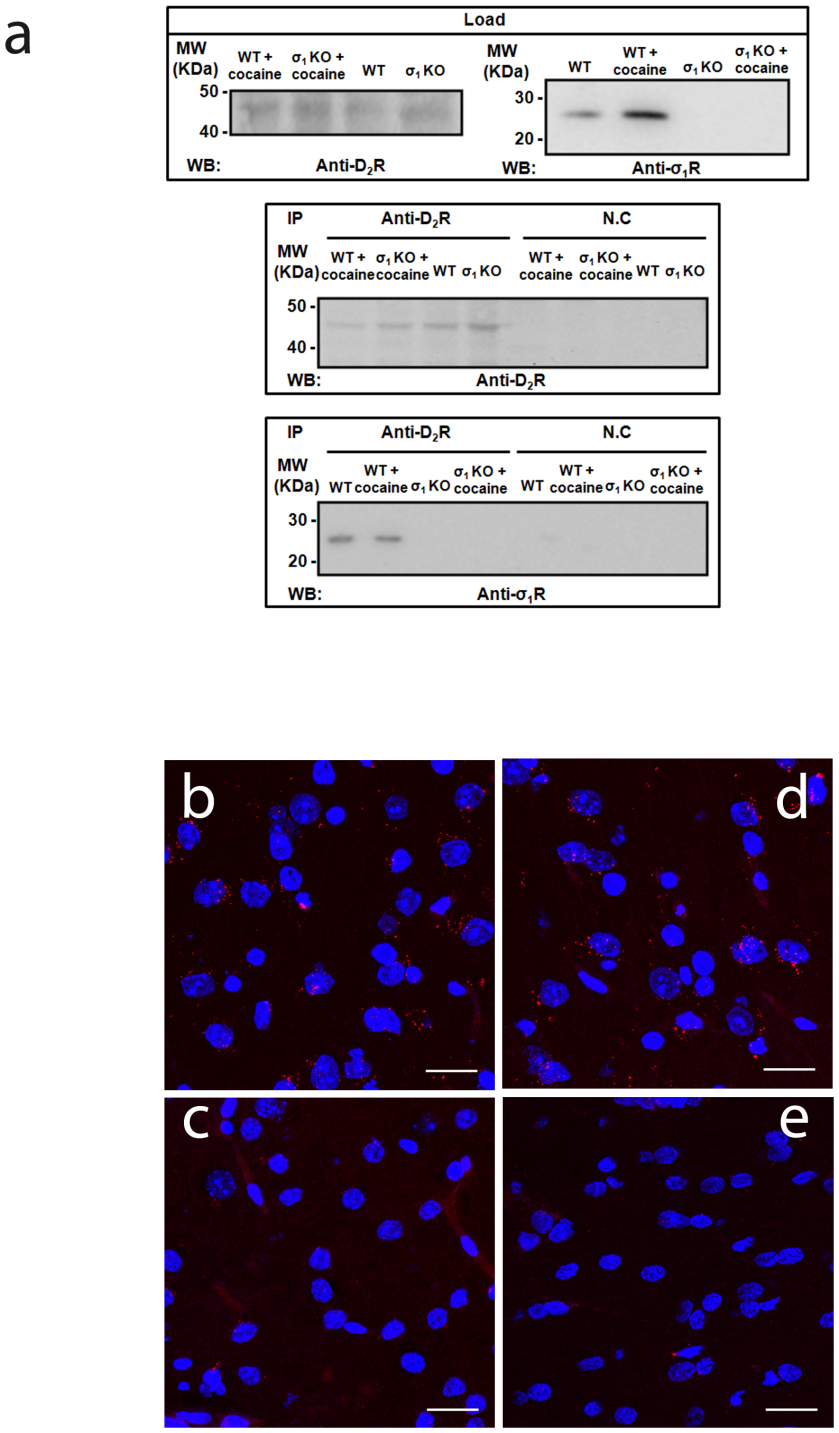
Expression of σ_1_-D_2_ receptor heteromers in the striatum. In (**a**) co-immunoprecipitation experiments are shown. Striatal slices from WT and KO mice were untreated or treated with 150 µM cocaine for 30 min. From slices solubilized striatal membranes (top panel) and immunoprecipitates with anti-D_2_ receptor antibody or anti-FLAG antibody as negative control (NC) (middle and bottom panels) were analyzed by SDS-PAGE and immunoblotted using mouse anti-D_2_ receptor antibody or mouse anti-σ_1_ receptor antibody. IP: immunoprecipitation; WB: western blotting; MW, molecular mass. In (**b to e**) Proximity Ligation Assay (PLA) was performed as indicated in Materials and Methods, using WT (b and d) or KO (c and e) mouse striatal slices not treated (b and c) or treated (d and e) with 150 µM cocaine for 30 min. σ_1_-D_2_ receptor heteromers were visualized as red spots around blue colored DAPI stained nucleus. Scale bar: 20 µm.

The BRET experiments and the signaling experiments are all suggestive of functional complexes that can lead to changes in D_2_ receptor function. However, all of these experiments were performed in transfected cells. To establish whether these complexes and their functional implications can be seen in tissue we obtained striatum from wild type (WT) and σ_1_ knockout (KO) mice. The striatum express D_2_ receptor containing neurons of the indirect motor pathway and is one of the key areas of the brain where cocaine imposes its effects. First we examined whether σ_1_-D_2_ receptor heteromers could be detected in native tissue. We performed Western blot experiments and found the expression of both receptors in the striatum of WT mice and the expression of D_2_ receptors but not σ_1_ receptors in the striatum of KO mice (Fig. 6a). Next we performed co-immunoprecipitation experiments and found the antibody against D_2_ receptor could indeed co-precipitate D_2_ receptors and σ_1_ receptor (Fig. 6a) in WT mice striatum treated or not with 150 µM cocaine. This co-precipitation was not observed when tissue from σ_1_ receptor KO animals was used (Fig. 6a). Although supportive of the BRET experiments above and highly suggestive of heteromers in striatum, we wanted to ensure that these complexes were not an artifact of the detergent solubilization. To test this we used the recently developed proximity ligation assay on slices of striatum from both WT and σ_1_ KO mice [42]. Using immunohistochemistry, we first checked the expression of σ_1_ receptors in WT animals but not in KO animals (Fig. S7) and the expression of D_2_ receptors in both WT and KO animals (Fig. S8). Next we performed the proximity ligation assay on striatal slices from WT animals. The slices were treated or not with 150 µM cocaine and as shown in Figure 6 (b and d) a red punctate fluorescent staining was observed, indicating both receptors are indeed in a complex in mice striatum in the presence or absence of cocaine. As a negative control we repeated this with only one of the two primary antibodies, and staining was not seen (Fig. S9). As expected, the red punctate fluorescent staining was not observed when the experiments were performed with striatal slices from σ_1_ KO mice (Fig. 6c and e). These data further support the existence of σ_1_-D_2_ receptor heteromers in the striatum.

### Cocaine Binding to σ_1_ Receptors Modulates the D_2_ Receptor Signaling in Mouse Brain Striatum

**Figure 7:**
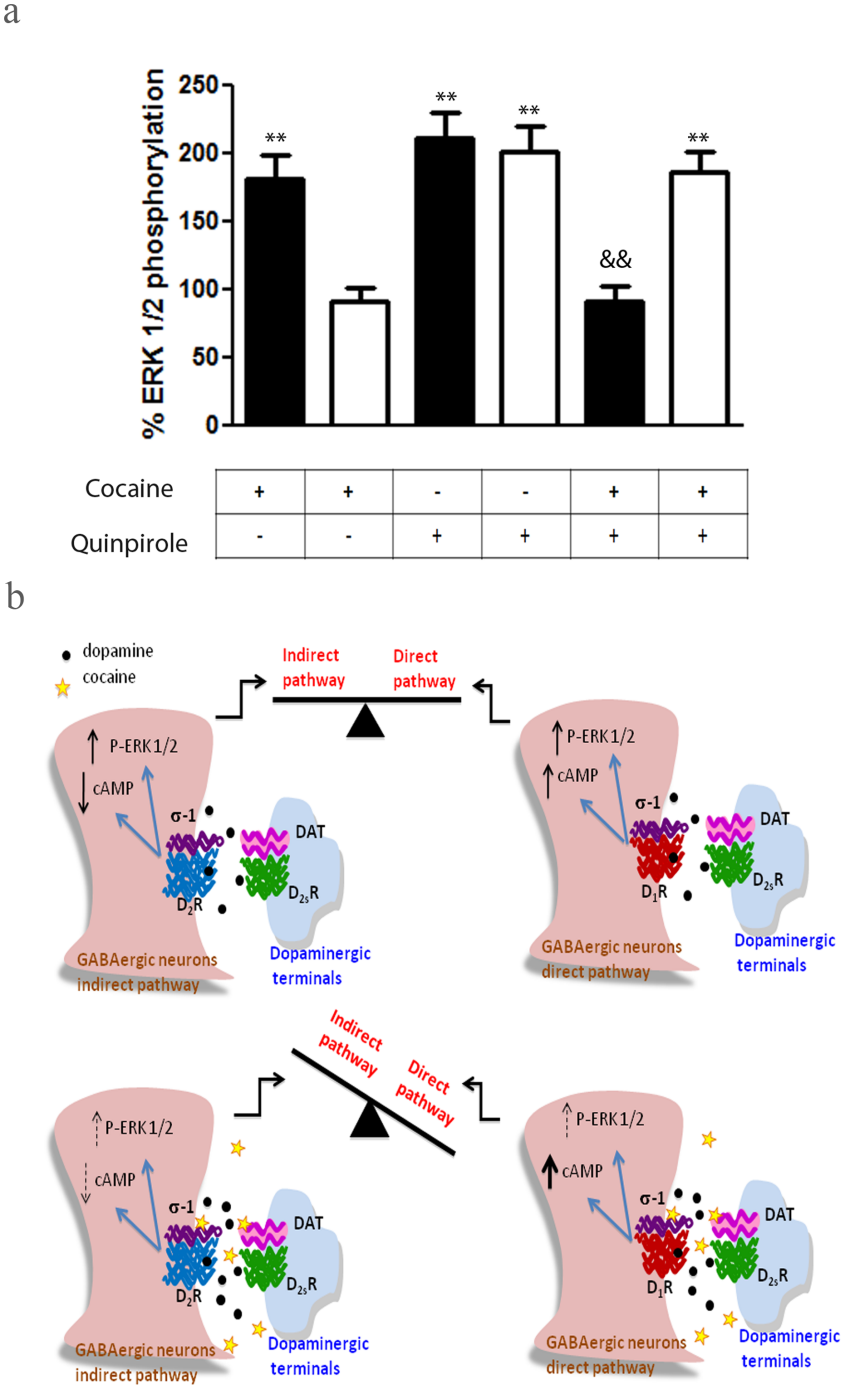
Negative cross-talk between cocaine and the D_2_ receptor agonist quinpirole on ERK 1/2 phosphorylation in mice striatum. In (**a**) WT (black bars) and σ_1_ receptor KO (white bars) mouse striatal slices were treated with 1 µM quinpirole for 10 min, with 150 µM cocaine for 30 min or with cocaine for 30 min and, during the last 10 min, with quinpirole. Immunoreactive bands from six slices obtained from five WT or five KO animals were quantified for each condition. Values represent mean ± SEM of percentage of phosphorylation relative to basal levels found in untreated slices. No significant differences were obtained between the basal levels of the WT and the σ_1_ receptor KO mice. Bifactorial ANOVA showed a significant (*p<0.05, **p<0.01, ***p<0.005) effect over basal. One-way ANOVA followed by Bonferroni post hoc tests showed a significant cocaine-mediated counteraction of quinpirole (^&^p<0.05, ^&&^p<0.01). In (**b**) a representative scheme summarizing the overall results is shown. Top images represent D_2_ and D_1_ receptors signaling in the indirect and direct striatal pathway neurons after dopamine binding. Bottom images represent the effect of cocaine increasing the dopamine by inhibiting dopamine transporters (DAT) and interacting with σ_1_ receptors within σ_1_-D_2_ and σ_1_-D_1_ receptor heteromers, changing the dopamine receptor signaling.

The above data provide strong evidence of σ_1_-D_2_ receptor heteromers in vivo but they do not say anything about the function of these complexes. We decided to test whether the negative cross-talk seen in signaling in transfected cells could also be found in the striatum. Striatum slices from WT and KO mice were tested for the effects of cocaine on ERK 1/2 phosphorylation. In co-transfected cells a strong and significant effect of cocaine was observed at 15 µM (see Fig. 5), a striatal level of the drug reached after pharmacologically significant doses of cocaine [43]. To allow diffusion into the tissue a ten-fold higher cocaine concentration, 150 µM, was then used to see clear effects in slices of mouse striatum. Both the D_2_ receptor agonist quinpirole (1 µM) and cocaine (150 µM) induced ERK 1/2 phosphorylation in striatal slices from WT mice after 10 min activation (Fig. S10) or after 30 min activation (Fig. 7a). More interestingly, in striatal slices of WT mice, the co-activation with quinpirole and cocaine blocked ERK 1/2 phosphorylation (Figs. 7a and S10). Thus, the negative cross-talk between σ_1_ and D_2_ receptors on MAPK signaling detected in cotransfected cells was also observed in striatal samples from WT mice, meaning that the same biochemical fingerprint seen in transfected cells was also found in WT mice. When similar experiments were performed in striatal slices from mice lacking the σ_1_ receptors, cocaine was unable to induce ERK 1/2 phosphorylation (Figs. 7a and S10) and quinpirole-induced ERK 1/2 phosphorylation was not modified by cocaine (Figs. 7a and S10). These results strongly support the existence of functional σ_1_-D_2_ receptor heteromers in the striatum and indicate that all detected cocaine effects are dependent on σ_1_ receptors expression.

## Discussion

The data presented in this paper lead to several major conclusions on the role σ_1_ receptors play in modulating D_2_ receptor upon cocaine exposure. First, D_2_ receptors can form heteromers with σ_1_ receptors, a result that is specific to D_2_ receptors as the other members of the D_2_-like family, D_3_ and D_4_ receptors, did not form heteromers. Second, these σ_1_-D_2_ receptor heteromers are found in mouse striatum and are functional. Third, σ_1_-D_2_ receptor heteromers consist of higher order oligomers with a minimal structure of σ_1_-σ_1_-D_2_-D_2_ receptor heterotetramers. Finally, cocaine, by binding to σ_1_-D_2_ receptor heteromers, inhibits downstream signaling in both cultured cells and in mouse striatum.

Cocaine intake elevates dopamine levels in the striatum, particularly in its more ventral part, the nucleus accumbens, which has been shown to be a preferential anatomical substrate for reward [44], [45]. Cocaine exploits the dopaminergic system to elicit part of its behavioral and cellular effects [14]. Earlier studies have suggested that the presynaptic dopamine transporter (DAT) is the primary target for cocaine effects [46]–[49]. However, not all cocaine effects are mediated by a dopamine increase derived by the cocaine inhibition of DAT. Indeed, cocaine interacts with many proteins, and it is now well established that cocaine interacts with σ_1_ receptors at physiologically relevant concentrations [50]–[55]. In fact, reducing brain σ_1_ receptor levels with antisense oligonucleotides attenuates the convulsive and locomotor stimulant actions of cocaine [56], [57] and antagonists for σ_1_ receptors have also been shown to mitigate the actions of cocaine in animal models [50], [58]. σ_1_ receptors are highly expressed in the brain [24], [59]. Within the caudate-putamen and nucleus accumbens (the dorsal and ventral parts of the striatum, respectively), brain regions that mediate the long-term effects of cocaine, it was demonstrated that repeated cocaine administration induces up-regulation of σ_1_ receptors, a process mediated by dopamine D_1_ receptors [60]. Indeed, we have demonstrated earlier the importance of the σ_1_ and D_1_ receptor interaction on the initial events upon cocaine exposure [16]. In addition, others have shown σ_1_ can modulate signaling of a different GPCR family [61]. Through σ_1_-D_1_ receptor heteromers, cocaine robustly potentiated D_1_ receptor-mediated adenylyl cyclase activation, providing a mechanism for D_1_ receptor-mediated effects of cocaine [16]. In addition to DAT and D_1_ receptors, our work here highlights the importance of σ_1_ receptors. Our data suggest that it is σ_1_ receptors that are able to directly modulate the normally balanced D_1_ and D_2_ pathways via receptor-receptor interactions.

The cocaine effect on σ_1_-D_2_ receptor heteromer signaling is in contrast with the cocaine effect on σ_1_-D_1_ receptor heteromer signaling described by Navarro et al [16]. In the last case, the D_1_ receptor-mediated activation of cAMP production was significantly increased by cocaine binding to a σ_1_ protomer in the σ_1_-D_1_ receptor heteromers, resulting in a cocaine-induced increase in cAMP production. The results here described and those described by Navarro et al [16], point to the scenario that is shown in Figure 7b, where cocaine selectively leads to increased dopamine-induced signaling through the cAMP pathway in D_1_ receptor-containing neurons and to depressed dopamine-induced inhibition of cAMP formation in D_2_ receptor-containing neurons. Simultaneously, cocaine alters the levels of the initial ERK 1/2 phosphorylation signaling induced by dopamine in both D_1_ receptor and D_2_ receptor-containing neurons. These findings suggest that cocaine exposure leads to a deregulation of a normally balanced D_1_/D_2_ dopamine receptor signaling (Fig. 7b). The balance of D_1_ and D_2_ inputs is designed to avoid addictive behavior, thus its disruption would have long term consequences. The data presented here support a key role of σ_1_ receptors in destabilizing this balance by increasing the D_1_ receptor-mediated cAMP production and dampening the D_2_ receptor signaling in σ_1_-D_2_ receptor heteromers, pushing the balance of inputs towards the D_1_ containing, pro-reward and motivating pathway. Our data is supported by the results described by Durieux and colleagues where they found that striatal D_2_R neurons can limit both locomotion and drug reinforcement and are organized in specific cell types [6], [8]. Luo et al [9], have found in vivo evidence for the existence of D_1_ and D_2_ receptor-mediated cellular effects of cocaine (D_1_ receptor-mediated increase in Ca^2+^ influx and D_2_ receptor-mediated decrease in Ca^2+^ influx, using in vivo optical microprobe Ca^2+^ influx imaging), with significantly slower dynamics of the effect mediated by D_2_ receptors. Taking into account our findings, the observations of Luo et al could in fact be linked with the signaling brake imposed by cocaine on the σ_1_-D_2_ receptor heteromer. Further, Ferraro et al. have found cocaine alone had no effect on striatal glutamate levels but when injected with a D_2_ ligand there were significant changes [62]. Xu et al have shown that a σ_1_ receptor ligand can reverse the effects of cocaine in rats strongly suggesting that blocking cocaine’s actions via σ_1_ receptor in σ_1_-D_2_ complexes could serve as an effective strategy to blunt the cellular signaling effects of cocaine [63]. Finally, Hiranita et al have shown that a combined strategy of blocking DAT and σ_1_ is effective at reducing cocaine self-administration. However, in a follow up study this same group shows that after cocaine self-administration σ_1_ receptor effects seem to be independent of dopamine pathways [54]. These are in line with our observations that the initial effects of cocaine disrupt the D_1_/D_2_ pathways. In summary, the results described here along with the highlighted previous studies support a model where the initial exposure to cocaine affects differently the direct (D_1_ containing) and indirect (D_2_ containing) pathways via σ_1_ receptor heteromers which may significantly influence dopaminergic neurotransmission.

## Supporting Information

Figure S1 **Chemical structure of compounds used.** a) cocaine, b) σ_1_ receptor agonist PRE084, c) σ_1_ receptor antagonist PD144418, d) D2 receptor agonist quinpirole, e) D2 receptor antagonist raclopride.
(TIF)

Figure S2 **Effect of σ_1_ receptor siRNA transfection on σ_1_ receptor expression.** Membranes from non-transfected HEK-293T cells (wt) or cells transfected with σ_1_ receptor siRNA (6.25 µg of oligonucleotides) or irrelevant oligonucleotides (oligo, 6.25 µg of oligonucleotides) were analyzed by SDS/PAGE and immunoblotted with the anti-σ_1_ receptor antibody. Values are mean ± SEM of three experiments. ***P<0.001 compared with non-transfected cells (one-way ANOVA followed by Bonferroni post hoc tests).
(TIF)

Figure S3 **Control CellKey label-free assays.** HEK-293T cells were stably transfected with the G_s_ protein-coupled adenosine A_2A_ receptor (a), the G_i_ protein-coupled adenosine A_1_ receptor (b) or untransfected (c) in 96 well Cell-Key plates. Impedance changes were measured upon addition of 10 nM CGS 21680 (A_2A_ receptor agonist) in (a), 10 nM CPA (A_1_ receptor agonist) in (b) or 50 nM thrombin (the agonist for the endogenous G_q_ protein-couples thrombin receptors) in (c). Plot shapes are consistent with the expected results for the respective G-proteins.
(TIF)

Figure S4 **σ_1_** **receptor agonist modulates the D_2_ receptor-mediated cAMP decreases.** cAMP production was determined in CHO cells stable expressing D_2_ receptors not transfected (black columns) or transfected (white columns) with siRNA corresponding to σ_1_ receptor (6.25 µg of oligonucleotides). Cells were stimulated with 5 µM forskolin in absence (100%) or presence of 1 µM quinpirole, 100 nM PRE084 alone or in combination. Percent of cAMP produced respect to forskolin treatment was represented. Results are as mean ± S.E.M from five independent experiments. Statistical significance was calculated by one way ANOVA followed by Bonferroni multiple comparison test; ***p<0.005 compared with forskolin-treated cells (100%) and ^&&^ p<0.01 compared with the corresponding only quinpirole-treated cells.
(TIF)

Figure S5 **Cocaine effect on ERK 1/2 phosphorylation in cells not expressing D_2_ receptors.** CHO cells were incubated with increasing cocaine concentrations for 30 min (a) or with 30 µM cocaine for increasing time periods (b). ERK1/2 phosphorylation is represented as percentage over basal levels (100%, non-treated cells). Results are mean ± SEM of three to four independent experiments performed in duplicate.
(TIF)

Figure S6 **Cocaine-induced σ_1_-D_2_ receptor heteromer-mediated ERK 1/2 phosphorylation in transfected cells.** CHO cells transfected with D_2_ receptor cDNA (1 µg, black bars) or cotransfected (white bars) with D_2_ receptor cDNA and σ_1_ receptor siRNA (6.25 µg of oligonucleotides) were incubated with increasing cocaine concentrations for 30 min (a), with 30 µM cocaine for increasing time periods (b), with increasing quinpirole concentrations for 10 min (c) or with 1 µM quinpirole for increasing time periods (d)**.** ERK1/2 phosphorylation is represented as percentage over basal levels (100%). Results are mean ± SEM of four to six independent experiments performed in duplicate. In all samples in (c) and (d) and samples without siRNA transfection in (a) and (b), Bifactorial ANOVA showed a significant (p<0.01) effect of cocaine or quinpirole over basal, and Bonferroni post hoc tests showed a significant counteraction of cocaine effect by siRNA (*p<0.05, **p<0.01 and ***p<0.005 compared with sample with the same treatment and with siRNA transfection).
(TIF)

Figure S7 **Expression of σ_1_ receptor in the striatum.** WT **(a)** or σ_1_ receptor KO **(b)** mouse striatal slices were processed for immunohistochemistry as indicated in Materials and Methods using an anti-σ_1_ antibody. Cell nuclei were stained with DAPI (blue). Scale bar: 20 µm.
(TIF)

Figure S8 **Expression of D_2_ receptor in the striatum.** WT **(a)** or σ_1_ receptor KO **(b)** mouse striatal slices were processed for immunohistochemistry as indicated in Materials and Methods using an anti-D_2_ antibody (green). Scale bar: 20 µm.
(TIF)

Figure S9 **Negative controls for in situ proximity ligation assays.** Negative controls for in situ proximity ligation assays (see Materials and Methods) were performed in WT mouse striatal slices incubated with only anti-σ_1_ **(a)** or anti-D_2_ **(b)** antibody as primary antibodies. Cell nuclei were stained with DAPI (blue). Scale bar: 20 µm.
(TIF)

Figure S10 **Negative cross-talk between cocaine and the D_2_ receptor agonist quinpirole on ERK 1/2 phosphorylation in mouse striatum.** WT (black bars) and σ_1_ receptor KO (white bars) mouse striatal slices were treated for 10 min with 1 µM quinpirole, with 150 µM cocaine or with both. Immunoreactive bands from six slices obtained from five WT or five KO animals were quantified for each condition. Values represent mean ± SEM of percentage of phosphorylation relative to basal levels found in untreated slices. No significant differences were obtained between the basal levels of the wild-type and the KO mice. Bifactorial ANOVA showed a significant (**p<0.01, ***p<0.005) effect over basal. One-way ANOVA followed by Bonferroni post hoc tests showed a significant cocaine-mediated counteraction of quinpirole (^&&&^P<0.005).
(TIF)
