# Supplementary figures and images for: Cocaine Inhibits Dopamine D2 Receptor Signaling via Sigma-1-D2 Receptor Heteromers
Source: PLoS One. 2013 Apr 18;8(4):e61245. doi: 10.1371/journal.pone.0061245 (PMC3630156; doi:10.1371/journal.pone.0061245)

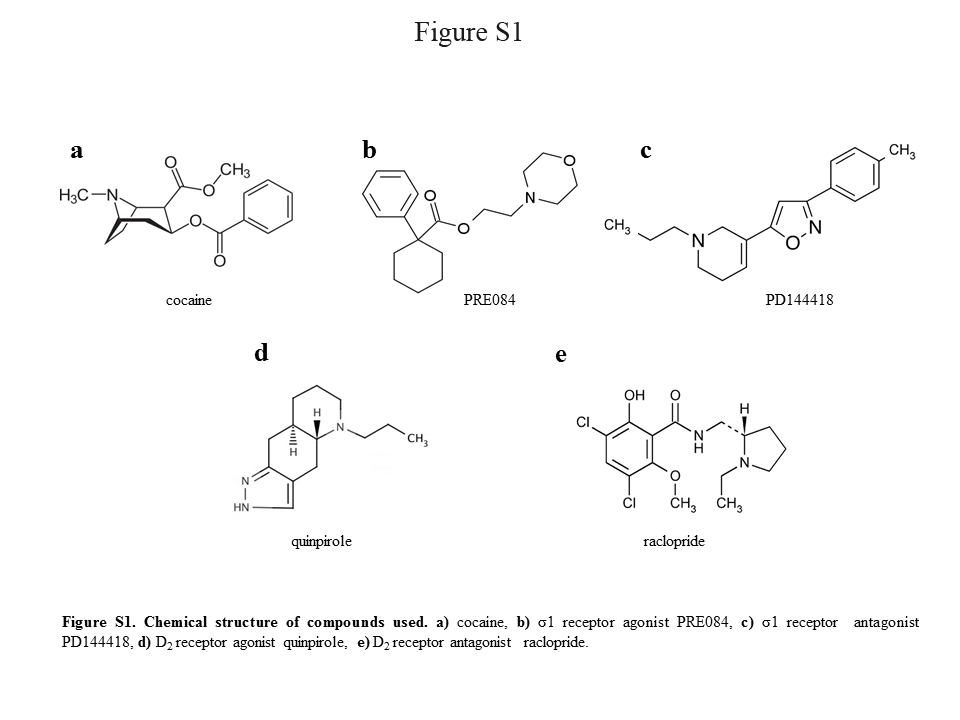

Supplement: Figure S1 — Chemical structure of compounds used. a) cocaine, b) σ1 receptor agonist PRE084, c) σ1 receptor antagonist PD144418, d) D2 receptor agonist quinpirole, e) D2 receptor antagonist raclopride. (TIF) [file pone.0061245.s001.tif]

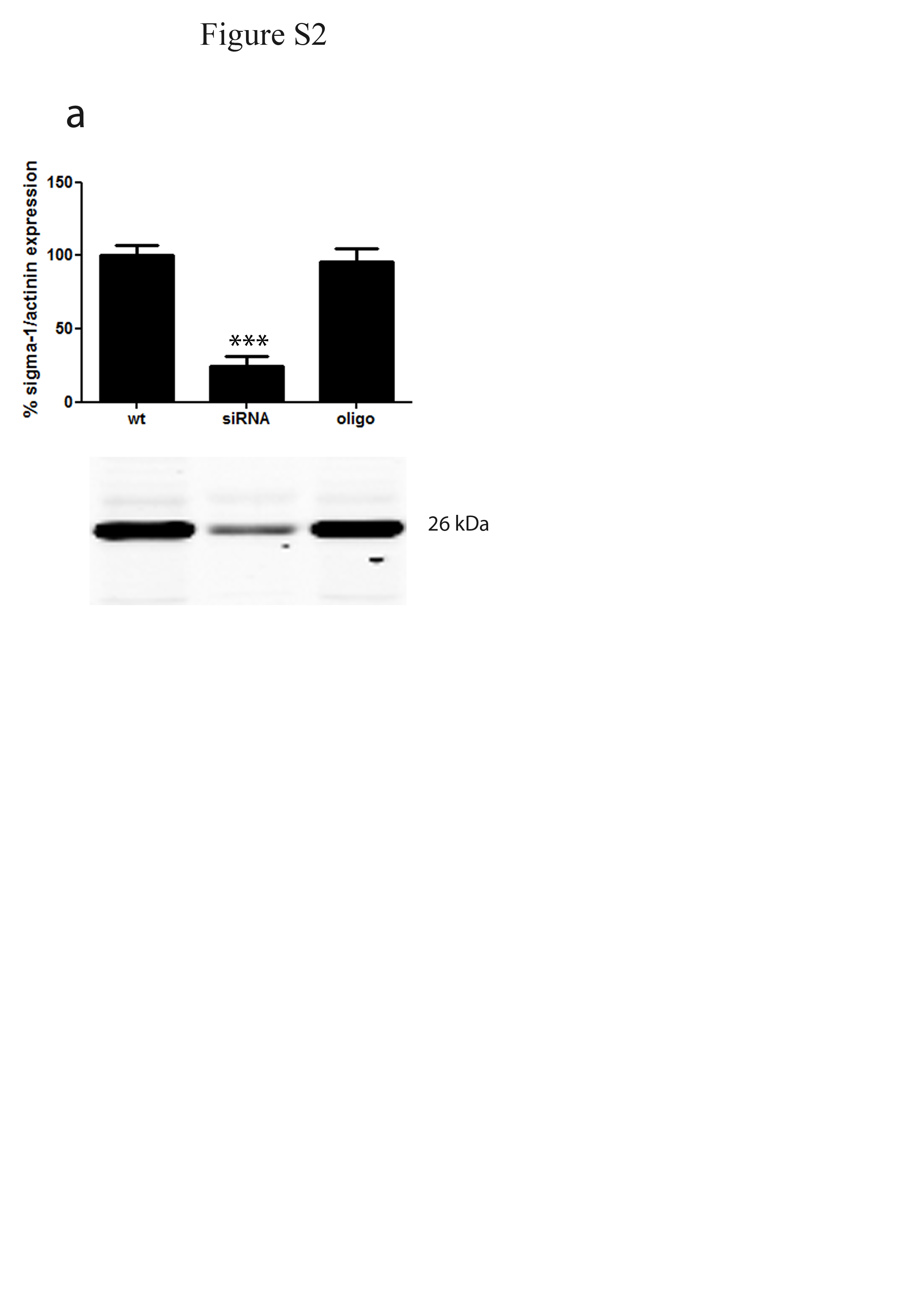

Supplement: Figure S2 — Effect of σ1 receptor siRNA transfection on σ1 receptor expression. Membranes from non-transfected HEK-293T cells (wt) or cells transfected with σ1 receptor siRNA (6.25 µg of oligonucleotides) or irrelevant oligonucleotides (oligo, 6.25 µg of oligonucleotides) were analyzed by SDS/PAGE and immunoblotted with the anti-σ1 receptor antibody. Values are mean ± SEM of three experiments. ***P<0.001 compared with non-transfected cells (one-way ANOVA followed by Bonferroni post hoc tests). (TIF) [file pone.0061245.s002.tif]

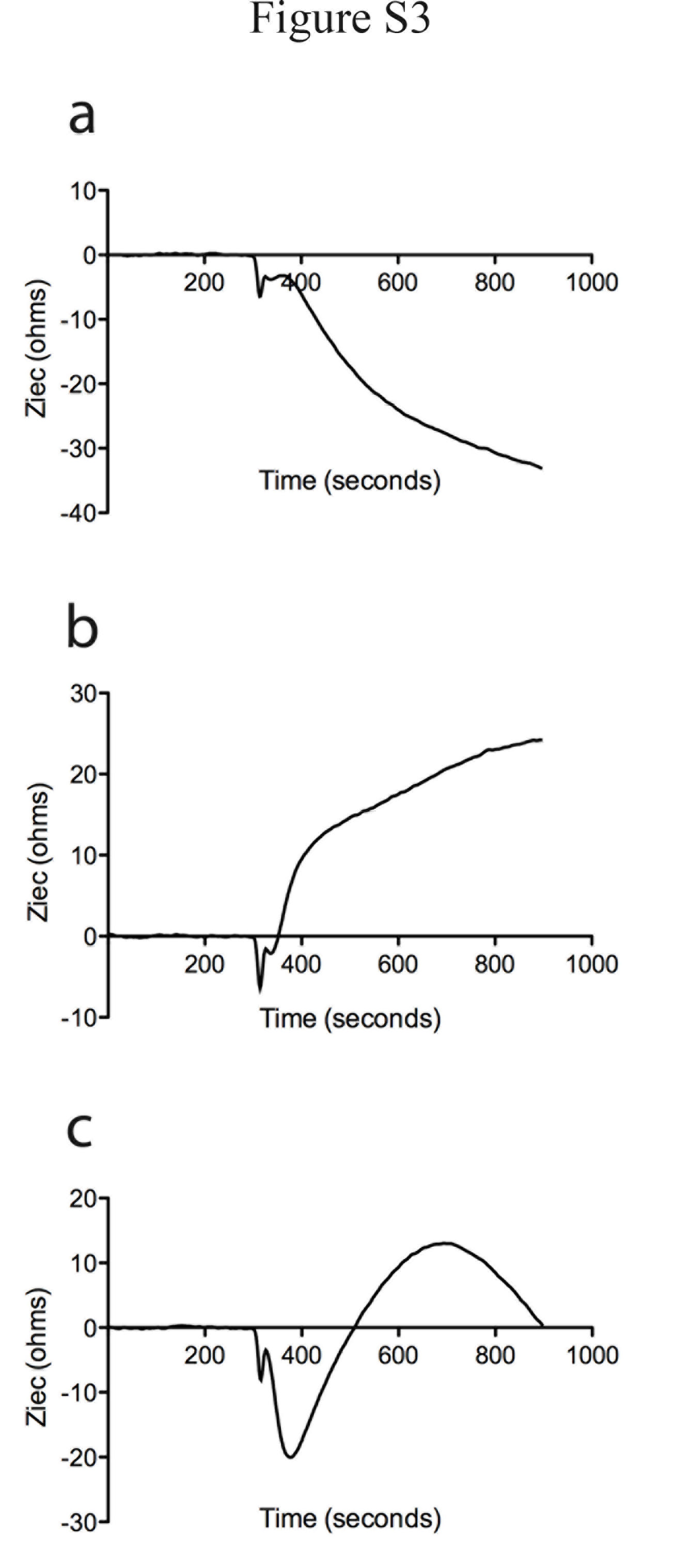

Supplement: Figure S3 — Control CellKey label-free assays. HEK-293T cells were stably transfected with the Gs protein-coupled adenosine A2A receptor (a), the Gi protein-coupled adenosine A1 receptor (b) or untransfected (c) in 96 well Cell-Key plates. Impedance changes were measured upon addition of 10 nM CGS 21680 (A2A receptor agonist) in (a), 10 nM CPA (A1 receptor agonist) in (b) or 50 nM thrombin (the agonist for the endogenous Gq protein-couples thrombin receptors) in (c). Plot shapes are consistent with the expected results for the respective G-proteins. (TIF) [file pone.0061245.s003.tif]

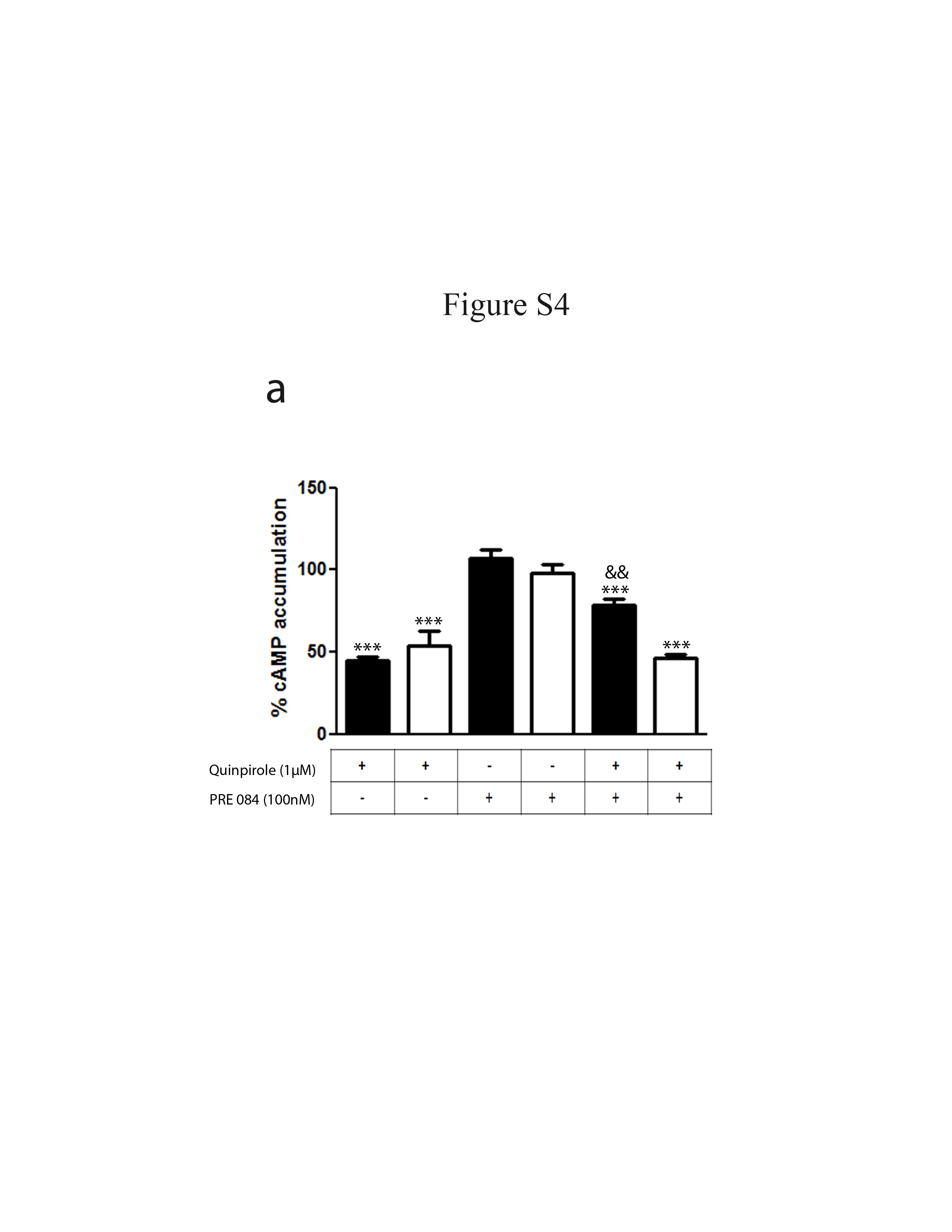

Supplement: Figure S4 — σ1 receptor agonist modulates the D2 receptor-mediated cAMP decreases. cAMP production was determined in CHO cells stable expressing D2 receptors not transfected (black columns) or transfected (white columns) with siRNA corresponding to σ1 receptor (6.25 µg of oligonucleotides). Cells were stimulated with 5 µM forskolin in absence (100%) or presence of 1 µM quinpirole, 100 nM PRE084 alone or in combination. Percent of cAMP produced respect to forskolin treatment was represented. Results are as mean ± S.E.M from five independent experiments. Statistical significance was calculated by one way ANOVA followed by Bonferroni multiple comparison test; ***p<0.005 compared with forskolin-treated cells (100%) and && p<0.01 compared with the corresponding only quinpirole-treated cells. (TIF) [file pone.0061245.s004.tif]

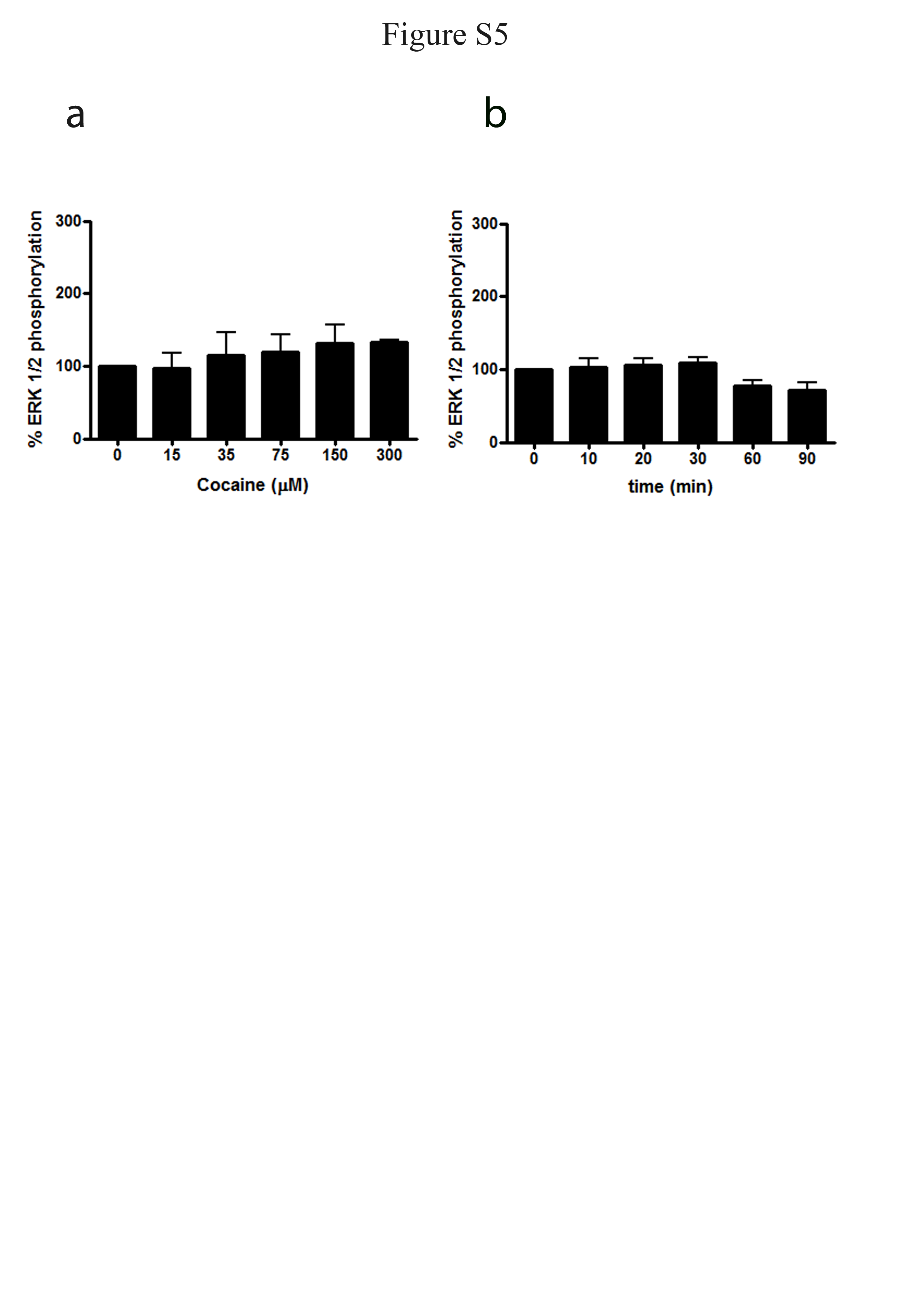

Supplement: Figure S5 — Cocaine effect on ERK 1/2 phosphorylation in cells not expressing D2 receptors. CHO cells were incubated with increasing cocaine concentrations for 30 min (a) or with 30 µM cocaine for increasing time periods (b). ERK1/2 phosphorylation is represented as percentage over basal levels (100%, non-treated cells). Results are mean ± SEM of three to four independent experiments performed in duplicate. (TIF) [file pone.0061245.s005.tif]

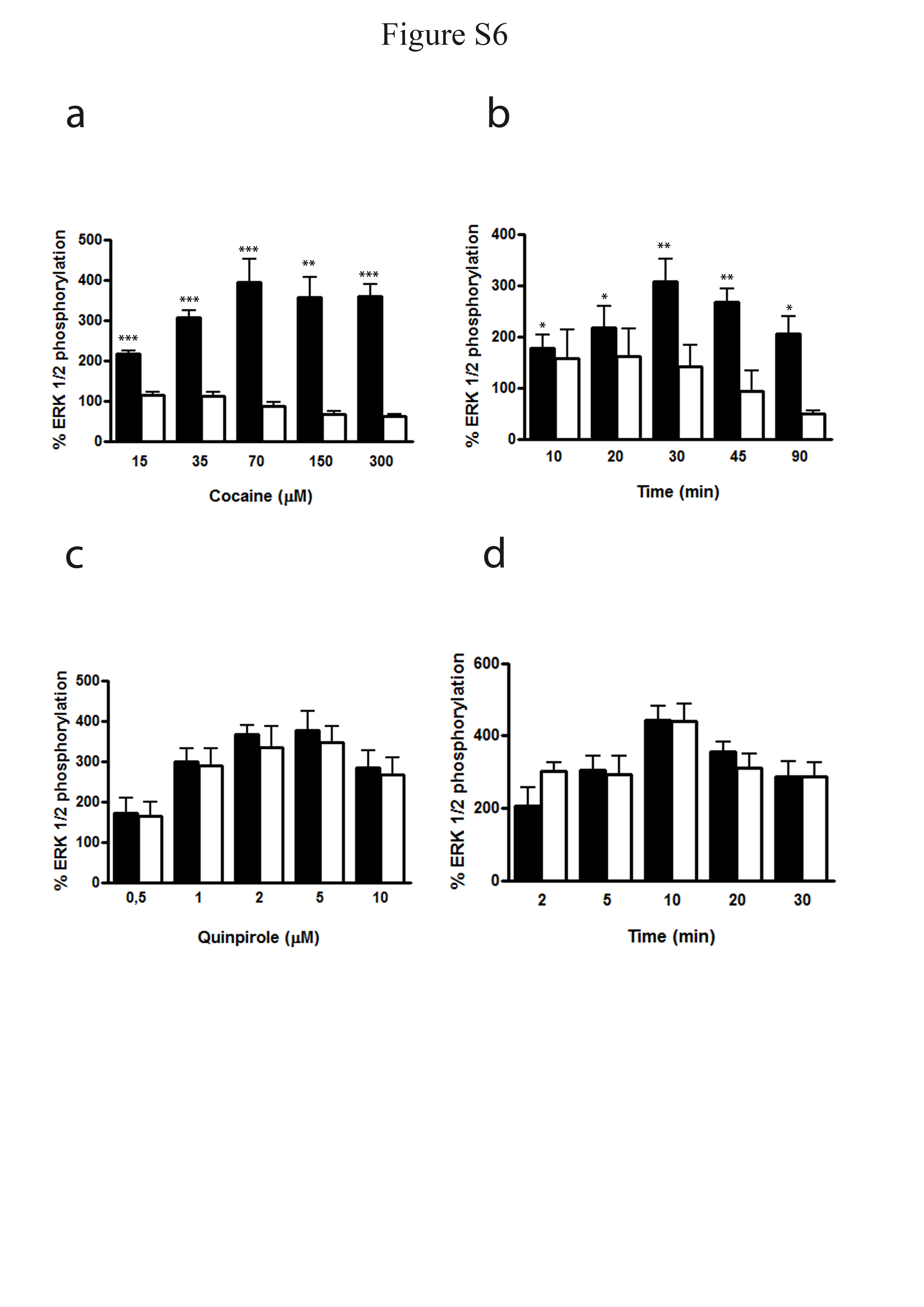

Supplement: Figure S6 — Cocaine-induced σ1-D2 receptor heteromer-mediated ERK 1/2 phosphorylation in transfected cells. CHO cells transfected with D2 receptor cDNA (1 µg, black bars) or cotransfected (white bars) with D2 receptor cDNA and σ1 receptor siRNA (6.25 µg of oligonucleotides) were incubated with increasing cocaine concentrations for 30 min (a), with 30 µM cocaine for increasing time periods (b), with increasing quinpirole concentrations for 10 min (c) or with 1 µM quinpirole for increasing time periods (d). ERK1/2 phosphorylation is represented as percentage over basal levels (100%). Results are mean ± SEM of four to six independent experiments performed in duplicate. In all samples in (c) and (d) and samples without siRNA transfection in (a) and (b), Bifactorial ANOVA showed a significant (p<0.01) effect of cocaine or quinpirole over basal, and Bonferroni post hoc tests showed a significant counteraction of cocaine effect by siRNA (*p<0.05, **p<0.01 and ***p<0.005 compared with sample with the same treatment and with siRNA transfection). (TIF) [file pone.0061245.s006.tif]

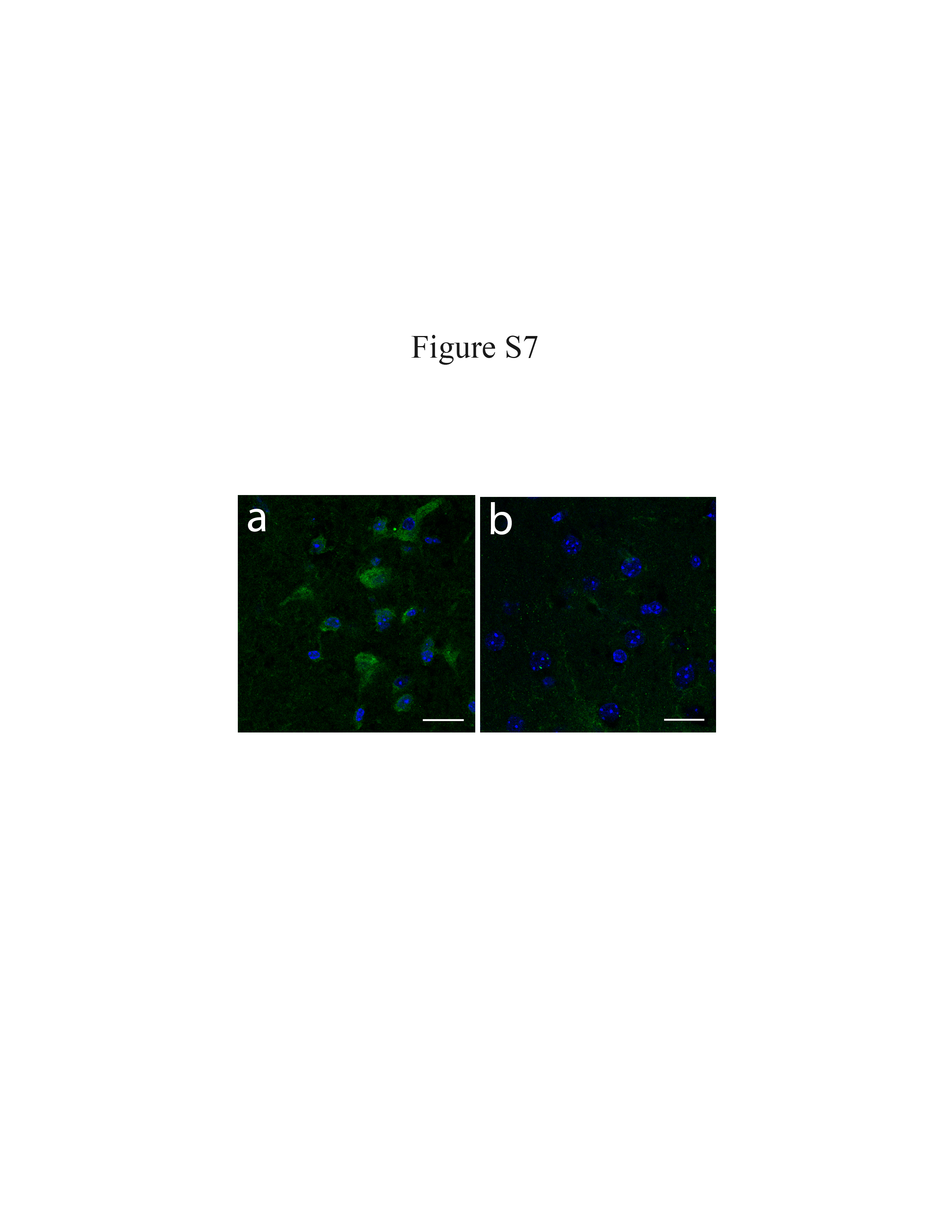

Supplement: Figure S7 — Expression of σ1 receptor in the striatum. WT (a) or σ1 receptor KO (b) mouse striatal slices were processed for immunohistochemistry as indicated in Materials and Methods using an anti-σ1 antibody. Cell nuclei were stained with DAPI (blue). Scale bar: 20 µm. (TIF) [file pone.0061245.s007.tif]

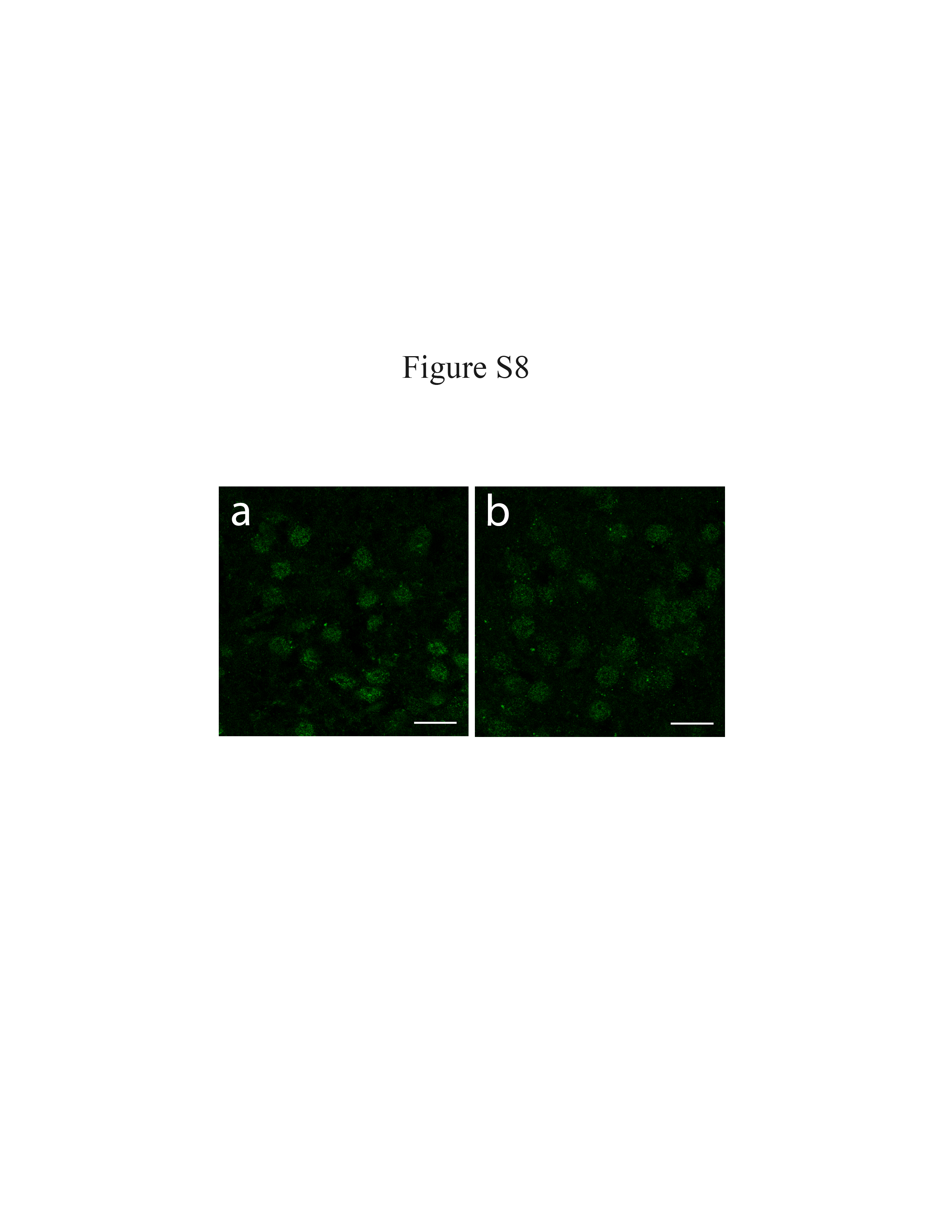

Supplement: Figure S8 — Expression of D2 receptor in the striatum. WT (a) or σ1 receptor KO (b) mouse striatal slices were processed for immunohistochemistry as indicated in Materials and Methods using an anti-D2 antibody (green). Scale bar: 20 µm. (TIF) [file pone.0061245.s008.tif]

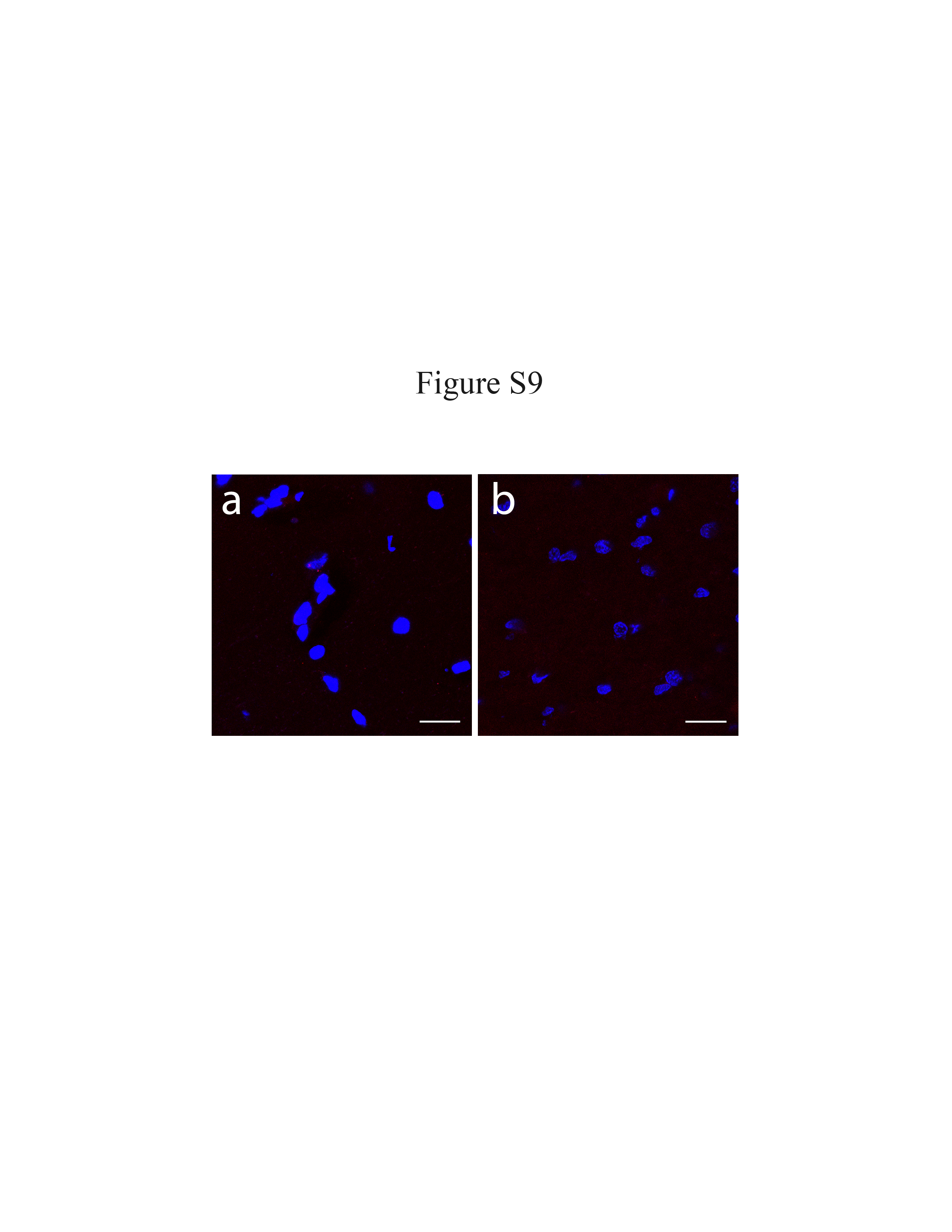

Supplement: Figure S9 — Negative controls for in situ proximity ligation assays. Negative controls for in situ proximity ligation assays (see Materials and Methods) were performed in WT mouse striatal slices incubated with only anti-σ1 (a) or anti-D2 (b) antibody as primary antibodies. Cell nuclei were stained with DAPI (blue). Scale bar: 20 µm. (TIF) [file pone.0061245.s009.tif]

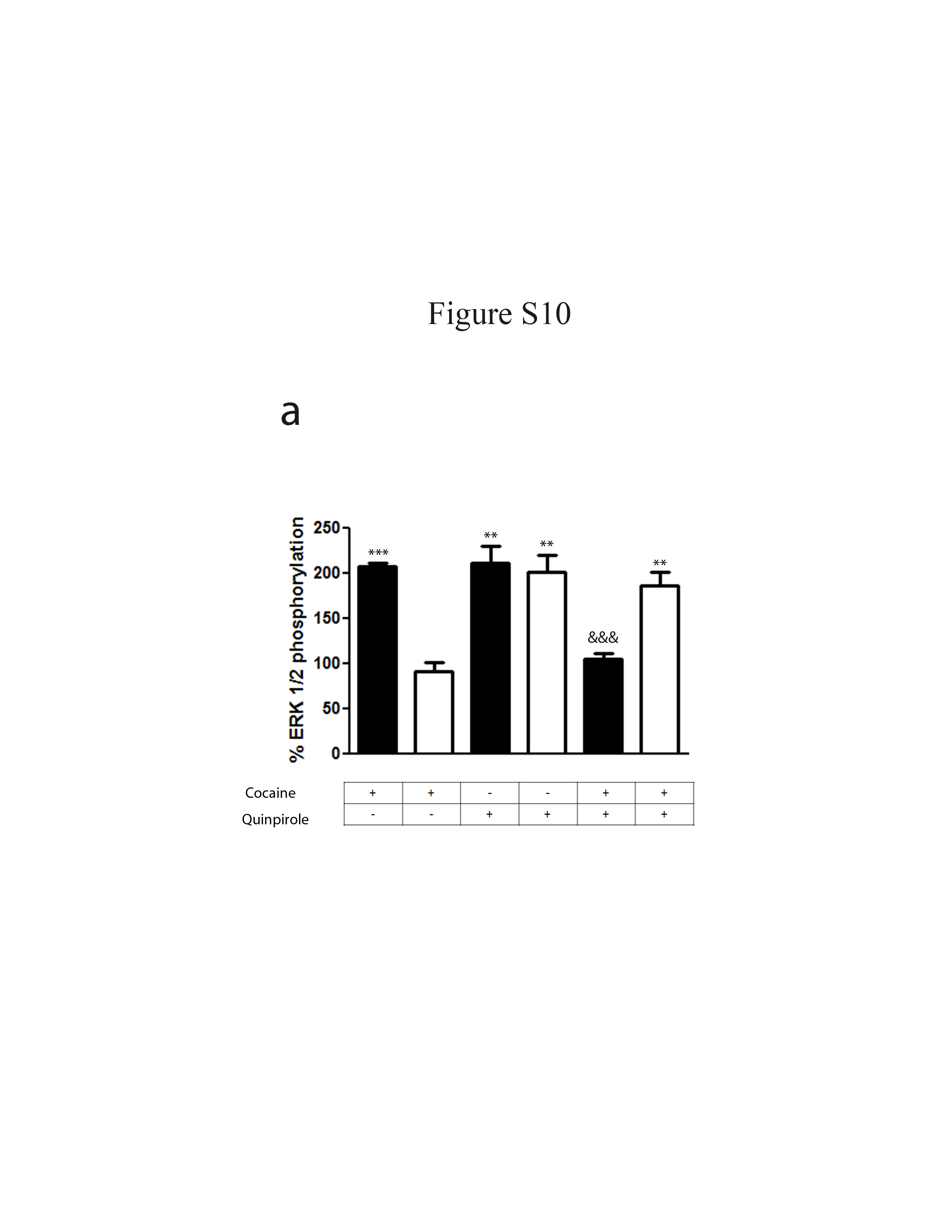

Supplement: Figure S10 — Negative cross-talk between cocaine and the D2 receptor agonist quinpirole on ERK 1/2 phosphorylation in mouse striatum. WT (black bars) and σ1 receptor KO (white bars) mouse striatal slices were treated for 10 min with 1 µM quinpirole, with 150 µM cocaine or with both. Immunoreactive bands from six slices obtained from five WT or five KO animals were quantified for each condition. Values represent mean ± SEM of percentage of phosphorylation relative to basal levels found in untreated slices. No significant differences were obtained between the basal levels of the wild-type and the KO mice. Bifactorial ANOVA showed a significant (**p<0.01, ***p<0.005) effect over basal. One-way ANOVA followed by Bonferroni post hoc tests showed a significant cocaine-mediated counteraction of quinpirole (&&&P<0.005). (TIF) [file pone.0061245.s010.tif]
